# Strategic Evaluation of the Traceless Staudinger Ligation for Radiolabeling with the Tricarbonyl Core

**DOI:** 10.3390/molecules26216629

**Published:** 2021-11-01

**Authors:** Constantin Mamat, Christian Jentschel, Martin Köckerling, Jörg Steinbach

**Affiliations:** 1 Helmholtz-Zentrum Dresden-Rossendorf, Institut für Radiopharmazeutische Krebsforschung, Bautzner Landstraße 400, D-01328 Dresden, Germany; c.jentschel@hzdr.de (C.J.); steinbach-joerg@web.de (J.S.); 2 Fakultät Chemie und Lebensmittelchemie, Technische Universität Dresden, D-01062 Dresden, Germany; 3 Institut für Chemie, Anorganische Festkörperchemie, Universität Rostock, Albert-Einstein-Straße 3a, D-18059 Rostock, Germany; martin.koeckerling@uni-rostock.de

**Keywords:** M(CO)_3_, ^99m^Tc, matched pair, theranostic approach, click chemistry, bioorthogonal

## Abstract

The traceless Staudinger ligation with its two variants is a powerful biorthogonal conjugation method not only for the connection of biomolecules, but also for the introduction of fluorescence- or radiolabels under mild reaction conditions. Herein, the strategic evaluation of the traceless Staudinger ligation for radiolabeling ^99m^Tc using the *fac*-[Tc(CO)_3_]^+^ core is presented. A convenient and high-yielding three-step synthetic procedure of dipicolylamine-based phosphanols as ligands for the mild radiolabeling was developed. The labeling was accomplished using a tricarbonyl kit and a ^99m^Tc-pertechnetate generator eluate showing 87% radiochemical conversion. The respective rhenium-based, non-radioactive reference compounds were synthesized using (Et_4_N)_2_[Re(CO)_3_Br_3_] as precursor. All products were analyzed by NMR, MS, and elemental analysis. Additional XRD analyses were performed.

## 1. Introduction

**Scheme 1:**
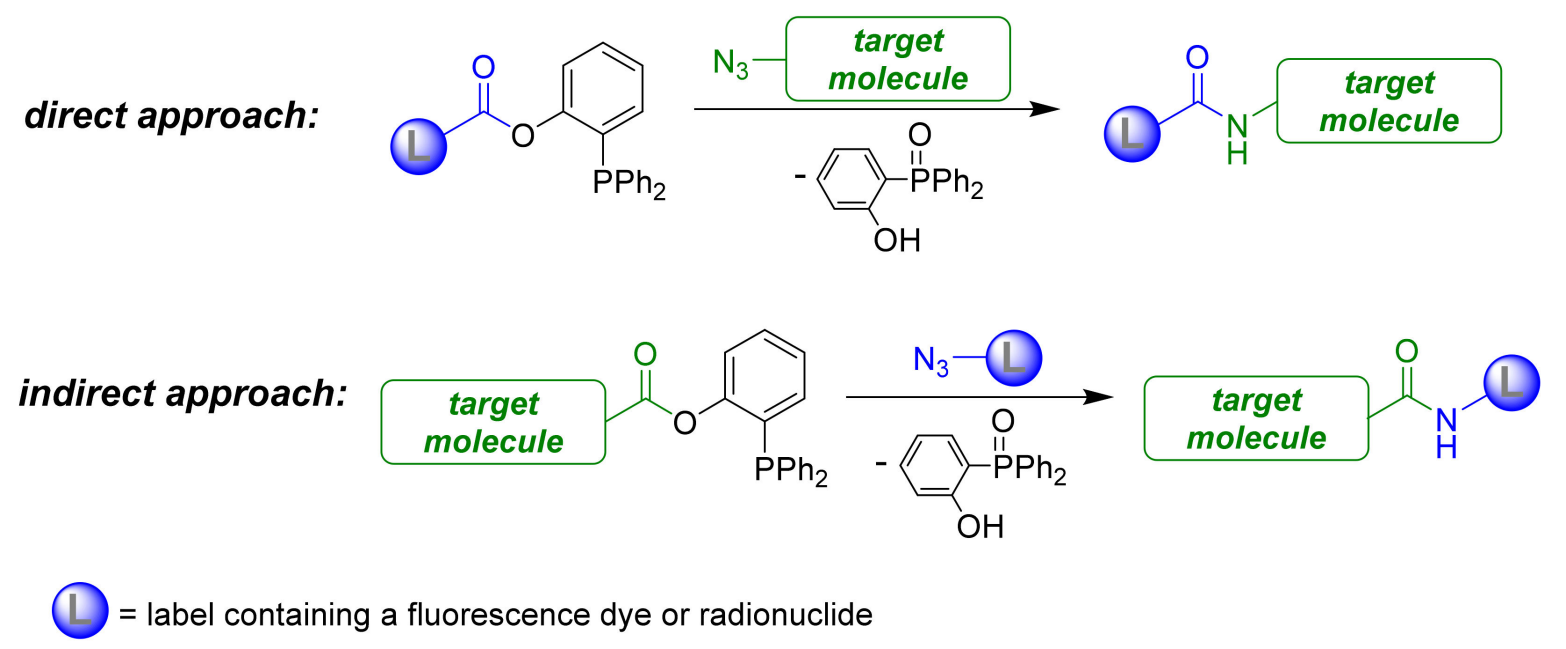
Direct and indirect approach of the traceless Staudinger Ligation.

The traceless Staudinger ligation belongs to the biorthogonal click conjugation reactions and is widely used, e.g., to connect small molecules to (bio)macromolecules like peptides, proteins, or carbohydrates, for cyclization processes to construct large sized lactams, or for the preparation of molecular rods [1,2,3,4,5]. In general, two variants of the traceless Staudinger ligation are described for (radio)labeling purposes [6,7]. Both approaches are shown in Scheme 1. The direct variant is based on a modified phosphane containing the (radio)label, which is reacted with an azide-containing biomolecule. The second, indirect variant, uses an azide-functionalized molecule bearing the (radio)label and a biomolecule, which is connected to the phosphane unit. Due to the smooth reaction conditions, the insertion of any label, such as fluorescent dyes [8] or radionuclides like fluorine-18 [9], iodine-131, or radiometals [7], is also possible without the need for a catalyst in contrast to other Cu-catalyzed click reactions.

In radiopharmacy, the convenient preparation of organometallic precursors bearing the *fac*-[Tc(CO)_3_]^+^ core [10,11] opens a convenient route to design new radiopharmaceuticals for single photon emission computed tomography (SPECT) [12]. Especially bio(macro)molecules like peptides, proteins, or antibodies can be labeled under mild conditions when using Tc- and Re-tricarbonyl precursors in combination with click chemistry methods [13]. This has been applied in the past using the “click-to-chelate” approach for instance [14,15,16], but never with the Staudinger ligation. The drawback of this Cu-click-conjugation method lies in the need of a copper catalyst, which must be removed prior to in vivo applications [16,17,18,19] to minimize toxicity.

To overcome this obstacle, the application of the traceless Staudinger ligation, known as a strong and powerful copper-free ligation method, was evaluated for radiolabeling with technetium-99m. For this purpose, phosphanes are required equipped with a chelator to insert the M(CO)_3_ core. The 2,2′-dipicolylamine (DPA) moiety [20,21,22], which is known to be an excellent tridentate chelator for the M(CO)_3_ core [23], was chosen and connected to the phosphanol skeleton. The corresponding ^nat^Re complexes were synthesized as non-radioactive reference and the procedure was transferred to radiolabeling chemistry using technetium-99m.

## 2. Results and Discussion

### 2.1. Preparation of Phosphanes

**Scheme 2:**
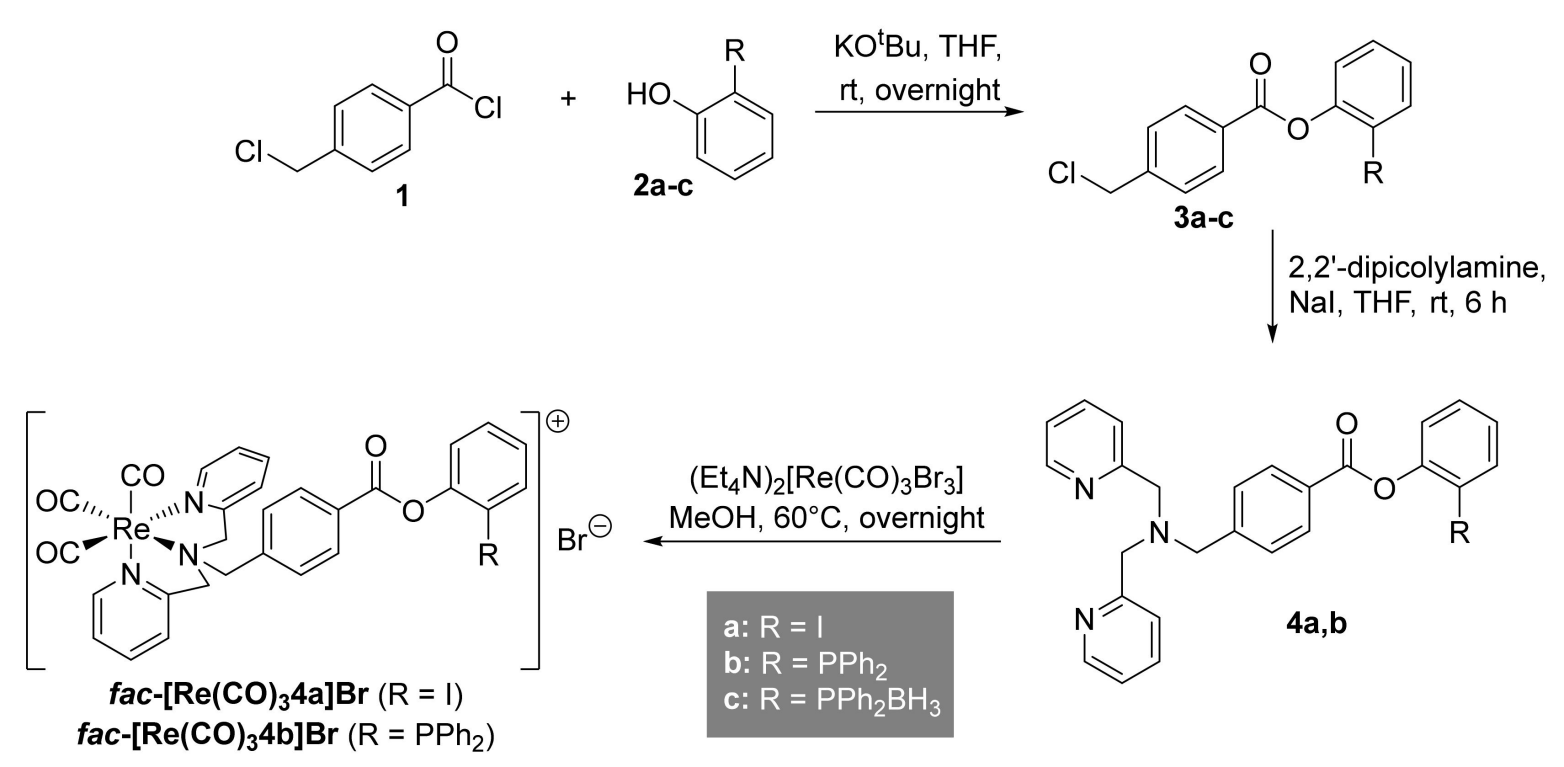
Synthesis of the 4-(chloromethyl)benzoate-building blocks **3a**–**c**, the 2,2′-dipicolylamine-containing ligands **4a,b** and their rhenium complexes ***fac-*[Re(CO)_3_4a]Br** and ***fac-*[Re(CO)_3_4b]Br**.

Azide-functionalized target molecules and label-containing phosphanes with benzoate moiety are required for the direct variant of the traceless Staudinger ligation (first line in Scheme 1). Two ways were elaborated in the past describing the functionalization of phosphanol **2b** or the preparation of functionalized 2-iodophenyl esters from **2a** followed by introduction of the phosphane residue [24,25]. Moreover, the dipicolylamine moiety, which is used as a chelating unit for the tricarbonyl core, was mandatory to connect to phosphanol skeleton by a benzoate linker. The synthesis route to the final phosphane-containing ligands with the dipicolylamine-chelating group is shown in Scheme 2.

In the first step, 4-(chloromethyl)benzoyl chloride (**1**) was reacted with 2-iodophenol (**2a**), phosphanol **2b**, and the borane-protected phosphanol **2c** to give the building blocks **3a**–**c** in high yields (97%, 84%, and 82%). Compound **3a** was synthesized to use the alternative route for the phosphane preparation, whereas **3c** was synthesized to avoid side-reactions like unwanted phosphonium-salt formation or oxidation of the phosphorus. Afterwards, **3a**–**c** were reacted with 2,2′-dipicolylamine in a nucleophilic substitution to yield the final DPA-ligands **4a** (86%) and **4b** (65%). Borane-containing compound **4c** could not be isolated due to decomposition. The last step involved the reaction of (Et_4_N)_2_[Re(CO)_3_Br_3_] as Re-tricarbonyl source [26,27,28] to the desired rhenium complexes ***fac-*[Re(CO)_3_4a]Br** and ***fac-*Re(CO)_3_4b]Br**, which are intended to be used as non-radioactive references for later radiolabeling with ^99m^Tc.

**Figure 1:**
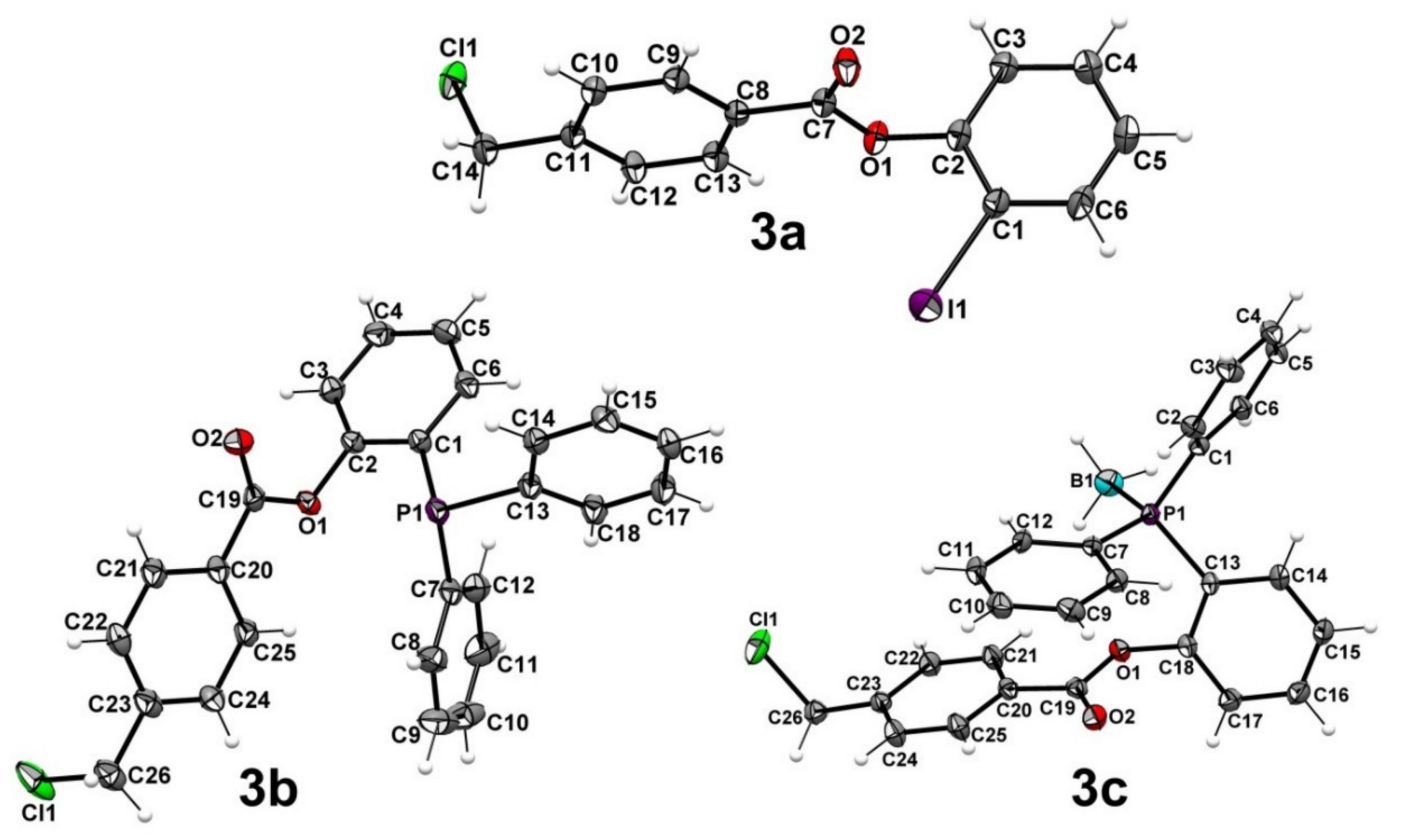
Molecular structures with atom labelling scheme of the 4-(chloromethyl)benzoate derivatives **3a**–**c**, ORTEP plots with 50% probability level (in the case of **3c** only one of the two symmetry independent molecules is shown).

**Figure 2:**
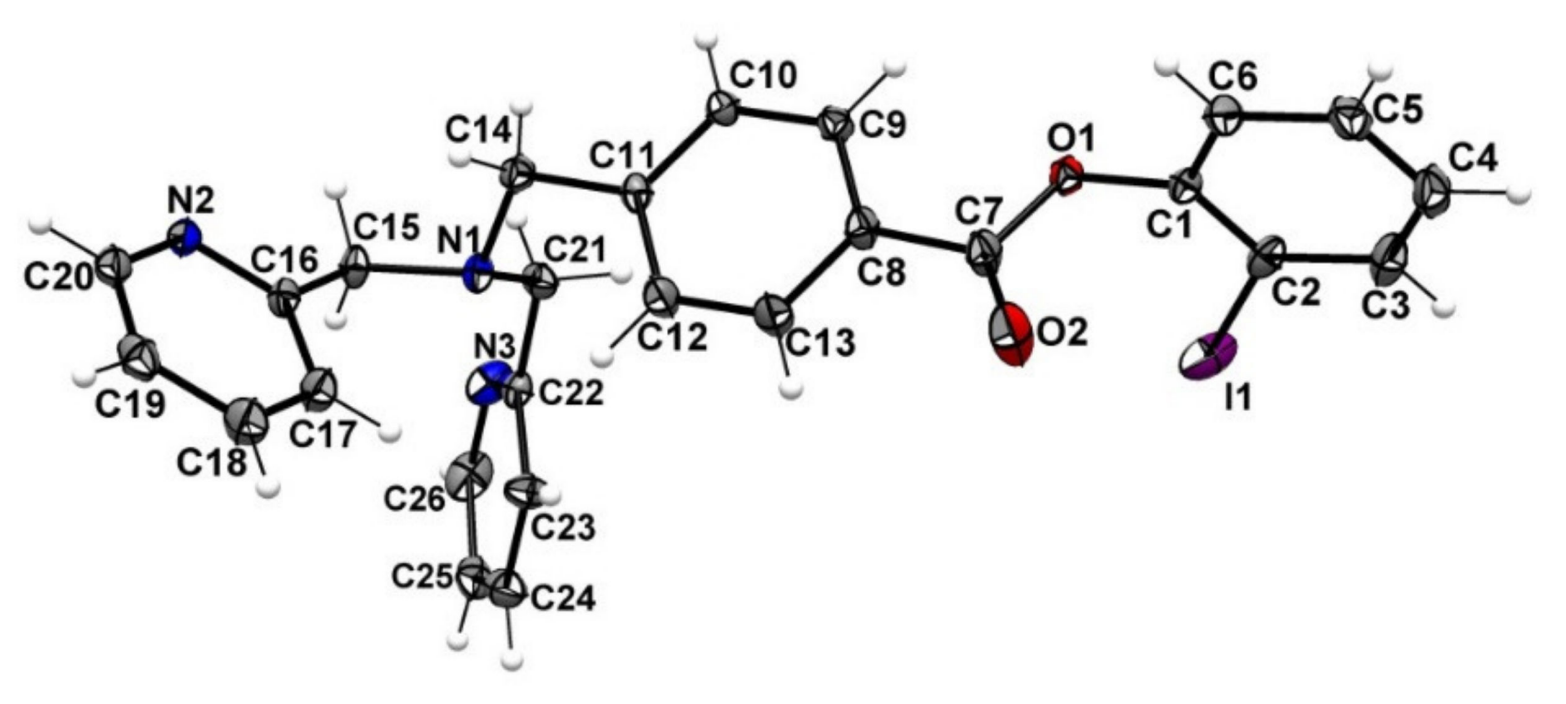
Molecular structure with atom labelling scheme of **4a**. ORTEP plot with 50% probability level (only one of the two symmetry independent molecules is shown).

The building blocks **3a**–**3c** and ligand **4a** were grown as single-crystals to establish their molecular structures from XRD analyses. The molecular structures of the compounds are presented in Figure 1 and Figure 2. All the bond lengths and angles are within their expected ranges. As expected, the average C–P–C angle in **3b** is significantly smaller (102.2°) than the ideal tetrahedral angle, whereas in **3c** the bonding angles around the phosphorous have an average of 109.4° with larger C–P–B and smaller C–P–C angles [24,25].

### 2.2. Preparation of the Non-Radioactive Rhenium-Reference Compounds

**Figure 3:**
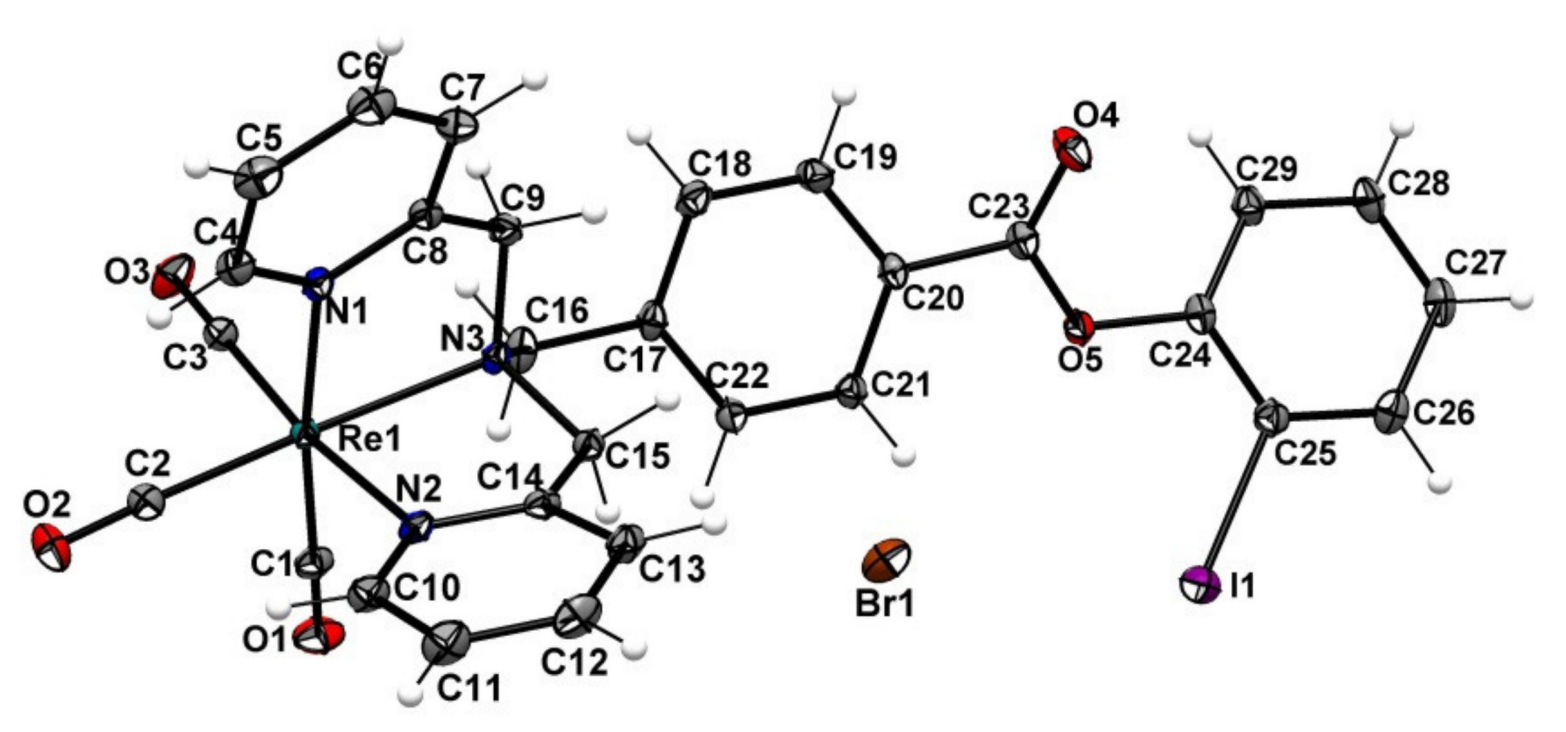
Structure of the rhenium complex ***fac-*[Re(CO)_3_4a]Br** (Ortep-Plot with 50% probability level, any co-crystallized methanol molecules are omitted for clarity).

Due to the lanthanide contraction, the ionic radii of Tc and Re are comparable, which leads to a similar coordination behaviour and complexation chemistry [12]. Rhenium compounds were classically used as non-radioactive reference for technetium-99m-tracers, or as therapeutic pendant when using rhenium-186/-188 [29,30,31] according to the theranostic concept [32,33]. For this purpose, ligand **4a** was reacted with (Et_4_N)_2_[Re(CO)_3_Br_3_] in methanol (last step in Scheme 2) to yield the desired rhenium complex ***fac-*[Re(CO)_3_4a]Br** in 95%. Its structure was confirmed by MS, NMR, and XRD analysis (Figure 3). Single crystals were obtained upon slow evaporation of the methanolic reaction mixture. Furthermore, in the ^1^H NMR spectrum, the facial arrangement of the DPA moiety induces a splitting of the methylene signals of the two picolyl units in ***fac-*[Re(CO)_3_4a]Br** into two doublets, which are found downfield-shifted at δ = 4.73 and 5.84 ppm (singlet of 3.86 ppm for ligand **4a**) with a coupling constant of ^2^*J* = 16.3 Hz. 

**Scheme 3:**
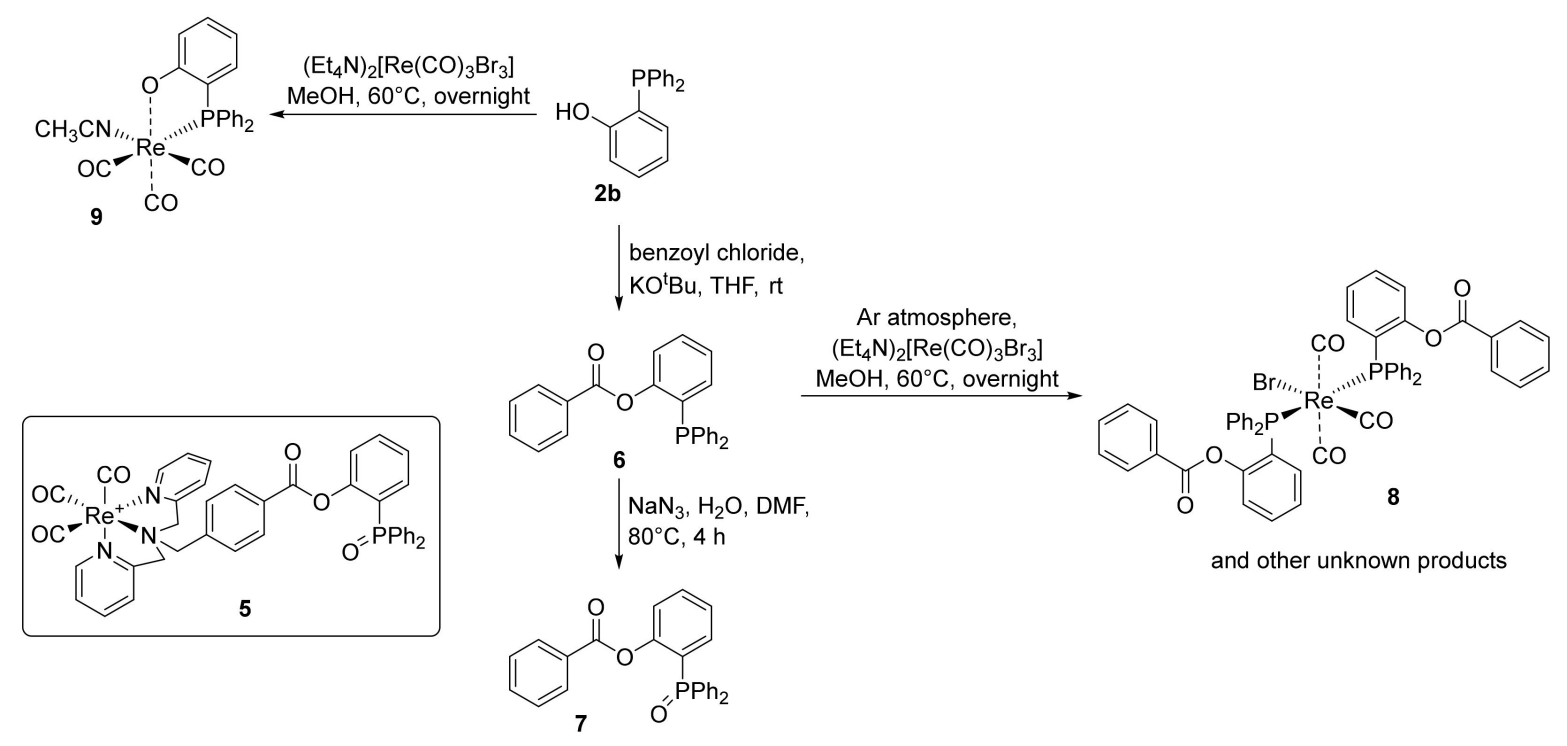
Synthesis of different phosphorous compounds **6**–**9** for structure elucidation of products obtained from the reaction of ligand **4b** with (Et_4_N)_2_[Re(CO)_3_Br_3_].

In contrast, when reacting **4b** with (Et_4_N)_2_[Re(CO)_3_Br_3_] under the conditions described for ***fac-*[Re(CO)_3_4a]Br**, a complex mixture of different complexation products was obtained, which were difficult to separate. The non-radioactive reference complex ***fac-*[Re(CO)_3_4b]Br**, which is required for later radiolabeling, was not found. For instance, compound **5** as oxidized species with bound *fac*-Re(CO)_3_ core could be identified (box in Scheme 3), but no ligation was possible anymore with **5**. A downfield-shifted signal of δ = 28.1 ppm was found in the ^31^P NMR and a peak at *m/z* = 880 was obtained from an ESI-MS analysis. To avoid, e.g., the oxidation of the phosphorous and the formation of other side products, the reaction was repeated under argon, with different solvents and temperatures, but without more success. In fact, a broad product mixture was obtained, because complexes of the *fac*-Re(CO)_3_ core are also able to be formed with the phosphorus of ligand **4b**. NMR spectra of all compounds can be found in the Supplementary Materials.

### 2.3. Attempt to Identify the Side Products from Complexation

**Figure 4:**
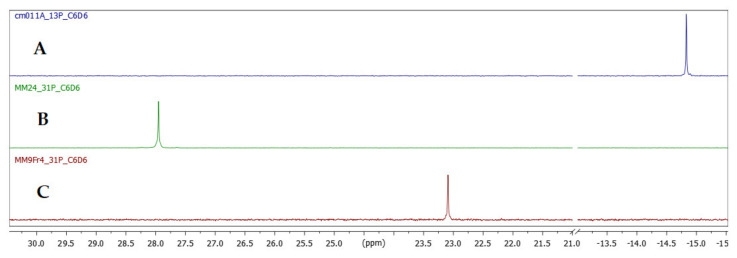
Comparison of ^31^P NMR spectra. (**A**): phosphane **6** (violet), (**B**): oxidized species **7** (green), and (**C**): phosphane complex **8** (red). All spectra were recorded in benzene-d_6_.

**Figure 5:**
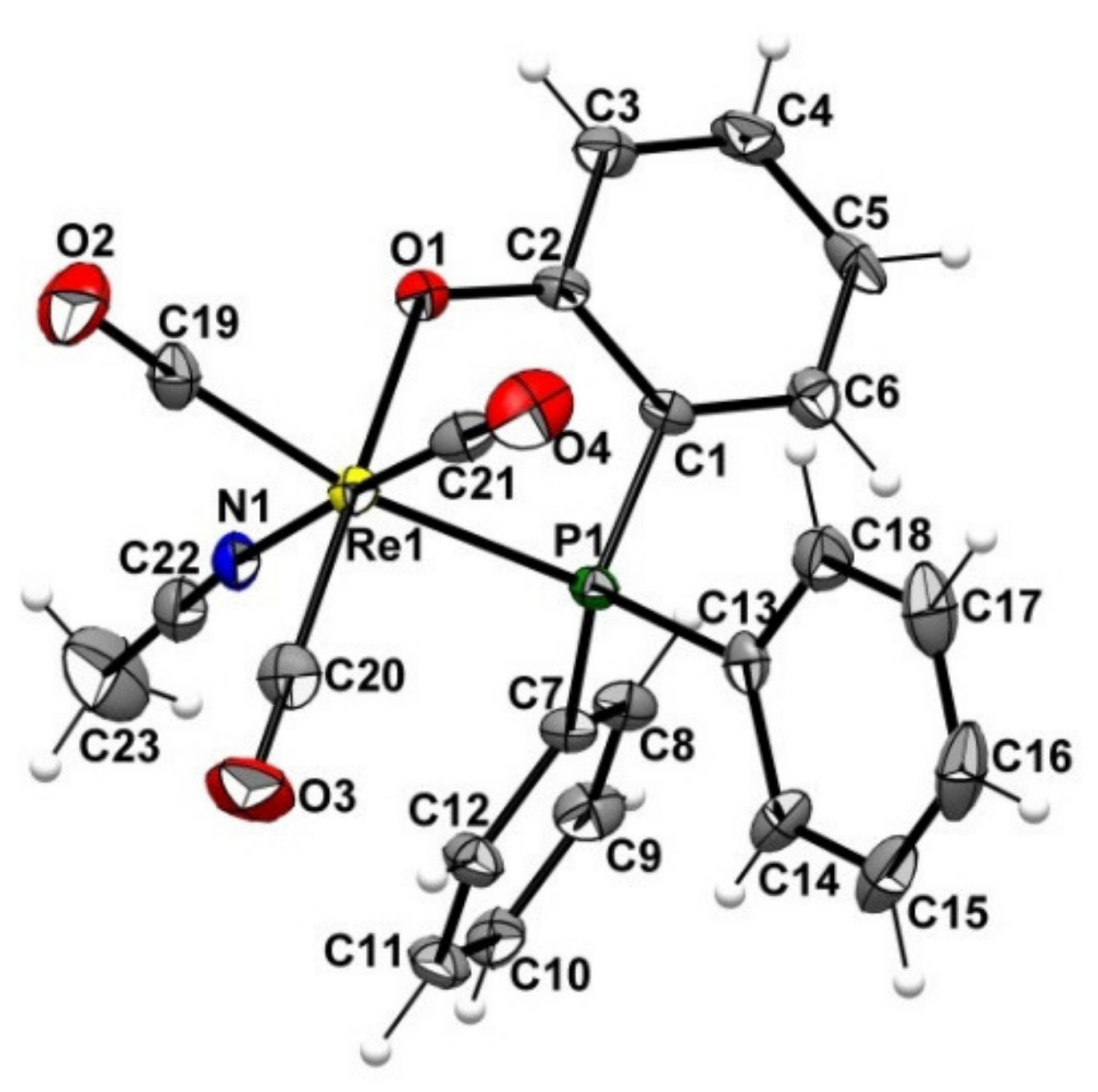
Molecular structure of the rhenium complex **9** (Ortep-Plot with 50% probability level, co-crystallized trichloromethane molecule is omitted for clarity).

In order to prove the complexation behavior of such compounds and to identify components of the above-mentioned product mixture, two different sample phosphanes **2b** and **6** [24] were chosen and treated under different reaction conditions with different starting materials as pointed out in Scheme 3. ^31^P NMR is an ideal tool to analyze such compounds and their behavior in terms of their oxidation state. Sample compound **6** was first oxidized to compound **7** to check the oxidation process during the complexation procedure. Next, phosphane **6** was reacted with (Et_4_N)_2_[Re(CO)_3_Br_3_] under argon giving a complex mixture, too. One of them was identified as complex **8**. A similar complex was described with PPh_3_ as ligand [34]. ^31^P NMR spectra of all synthesized compounds were compared (Figure 4). A signal of δ = −14.9 ppm was determined for phosphane **6** whereas a downfield-shifted signal of δ = 28.0 ppm was found for the oxidized species **7**. Furthermore, a downfield-shifted signal of δ = 23.1 ppm was found for complex **8**, indicating a coordinative bond of the phosphorus to the rhenium atom. An ESI-MS analysis of **8** revealed that phosphane **6** is twice coordinated as monodentate ligand (*m/z* = 1118). To further confirm the reaction behavior of the used phosphanes and to further elucidate the complexation pattern, (Et_4_N)_2_[Re(CO)_3_Br_3_] was directly reacted with phosphanol **2b** yielding complex **9**. Interestingly, ligand **2b** is bidentate [35] in Re-complex **9** in contrast to ligand **5**. This finding is supported by an XRD analysis of single crystals grown from complex **9** (Figure 5).

In both structurally characterized rhenium complexes, the metal atoms are coordinated in a distorted octahedral geometry. The coordination environment in ***fac-*[Re(CO)_3_4a]Br** (Figure 3) consist of three carbonyl-carbon atoms and three nitrogen atoms, of which two are aromatic nitrogen atoms (pyridine, N1 and N2) and one aliphatic (N3). As expected, a carbonyl ligand is bound on the opposite (*trans*) site of each nitrogen atom. N3 has a significantly longer bond to Re1 (2.233(1) Å) than N1 and N2 (2.169(2) and 2.171(2) Å). The Re–C (carbonyl) bond lengths are found in a narrow range of 1.914(2) to 1.831(2) Å. Because of these differences in bond lengths and due to steric restrictions of ligand **4a**, the *cis* arranged bond angles around Re1 differ from ideally 90° and range from 78.05(5)° to 97.88(7)°. In complex **9** (Figure 5), the Re1 atom is also octahedrally coordinated. The deprotonated bidentate ligand **2b** is coordinated via the oxygen and the phosphorus atom to the central Re atom. The remaining coordination sites are occupied with the carbon atoms of three bound carbonyl ligands and additionally one nitrogen atom of an acetonitrile ligand. As in ***fac-*[Re(CO)_3_4a]Br**, a *trans* arrangement of the different ligands is observed. The longest Re–ligand distance is found for P1, the next shortest for N1 and the shortest for O1. The lengths of the *trans*-Re–C (carbonyl) bonds follow the same order. The longest is found for C19 (1.952(6) Å, trans to P1), the next shorter for C21 (trans to N1, 1.915(6) Å), and the shortest for C20 (1.906(6) Å, trans to O1).

### 2.4. Radiolabeling with Technetium-99m

For the following radiolabeling with technetium-99m, a two-step procedure was applied, first to generate the ^99m^Tc(CO)_3_ species from the ^99m^Tc-pertechetate solution using a tricarbonyl kit and secondly to perform the final complexation with the DPA-containing ligands [36]. For this purpose, approximately 500 MBq of [^99m^Tc]TcO_4_^−^ in 1 mL saline was added to the kit and heated at 100 °C for 30 min. The resulting *fac*-[[^99m^Tc]Tc(CO)_3_(H_2_O)_3_]^+^ complex was reacted with ligands **4a,b** in a MES buffer/ethanol mixture at pH 6.2 for 30 min at 100 °C. After cooling to rt, purification was done using cartridge separation. 

**Figure 6:**
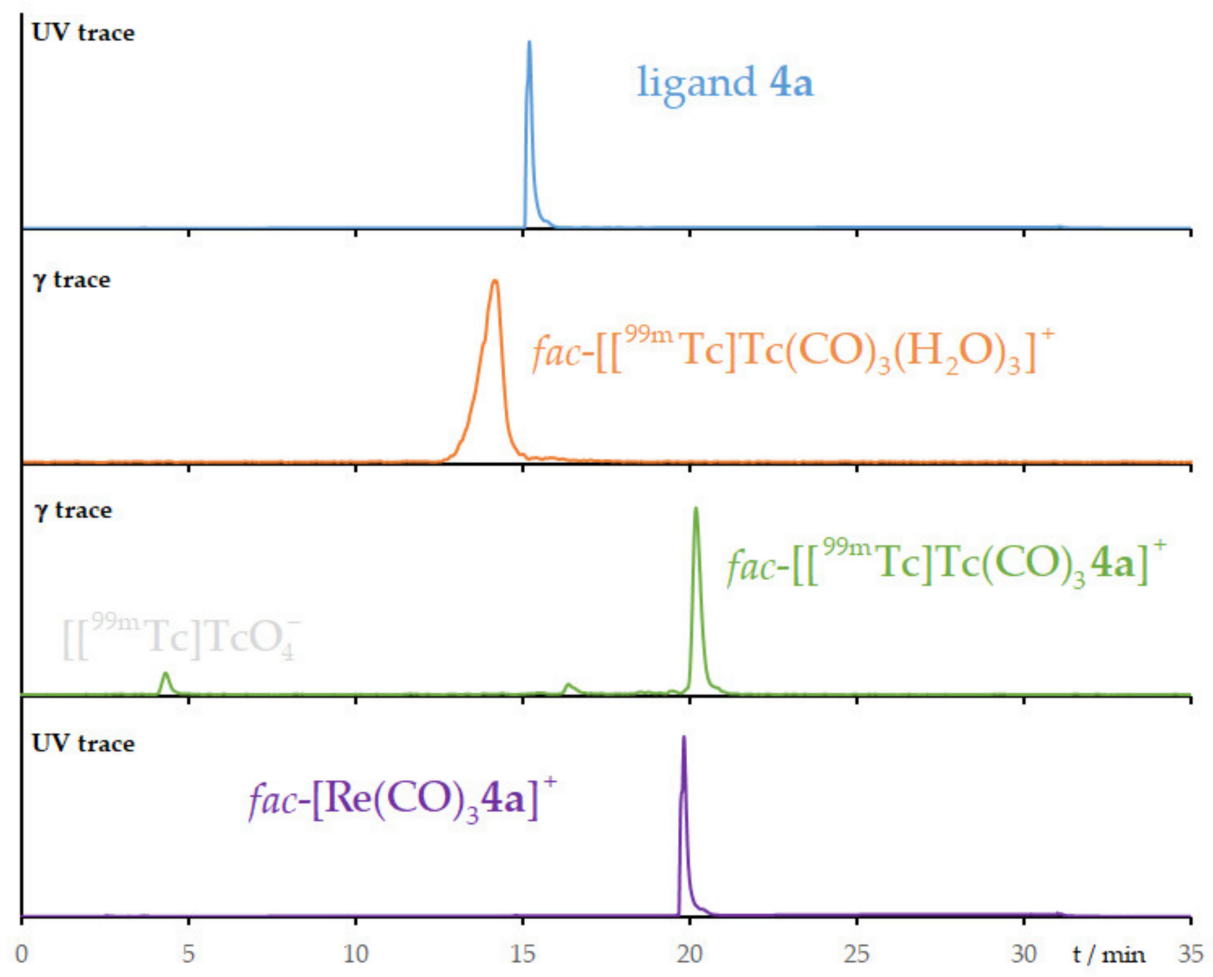
HPLC chromatograms (UV-/γ-traces) of ligand **4a** (blue, t_R_ = 15.2 min), the ^99m^Tc-tricarbonyl precursor (orange, t_R_ = 14.1 min), ***fac-*[[^99m^Tc]Tc(CO)_3_4a]^+^** (green, t_R_ = 20.2 min), and the non-radioactive Re-reference ***fac-*[Re(CO)_3_4a]Br** (violet, t_R_ = 19.8 min).

In the case of radiolabeling with ligand **4a**, complex ***fac-*[[^99m^Tc]Tc(CO)_3_4a]^+^** was obtained showing a peak at t_R_ = 20.2 min in the radio-HPLC chromatogram with 100% conversion of reduced [[^99m^Tc]Tc(CO)_3_(H_2_O)_3_]^+^ (t_R_ = 14.1 min). Two by-products were found, either at t_R_ = 4.2 min (9%) or at t_R_ = 16.4 min (4%), which are identified as back-oxidized TcO_4_^–^ and an unknown ^99m^Tc-species, respectively. Both by-products were removed after simple purification using a LiChrolut RP-18 cartridge. The identification of the ^99m^Tc-complex was confirmed with the respective rhenium complex ***fac-*[Re(CO)_3_4a]Br** (t_R_ = 19.8 min). Additionally, the UV signal of ligand **4a** was found at t_R_ = 15.2 min. All chromatograms for this radiolabeling procedure are shown in Figure 6. Details of the radiolabeling procedure can be found in the Supplementary Materials.

**Figure 7:**
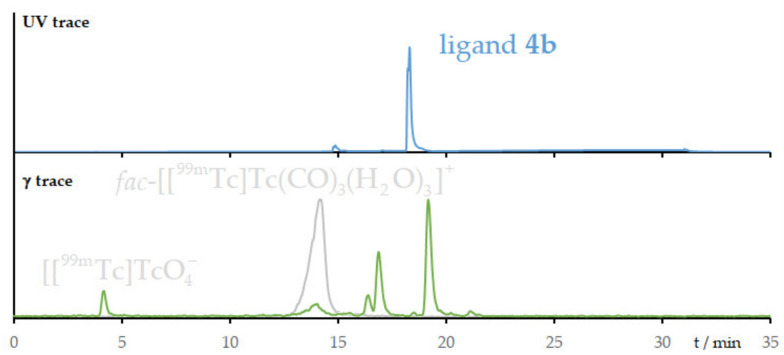
(Radio) HPLC chromatograms of ligand **4b** (blue, t_R_ = 15.2 min), the ^99m^Tc-tricarbonyl precursor (gray, t_R_ = 14.1 min), and the reaction mixture after radiolabeling (green).

In the case of radiolabeling with ligand **4b**, minimum five peaks were found in the radio-HPLC chromatogram (Figure 7) independent of the change of reaction parameters like solvent (ACN, THF, DMF, DMSO, EtOH), temperature (25–100 °C) and labeling time. Beside the known by-product peaks at t_R_ = 4.1 and 16.4 min, two main signals at 16.9 min (24%) and at 19.2 min (45%) occurred together with two by-products at 14.0 min (11%, remaining ^99m^Tc-precursor) and at 16.4 min (7%). At this point, it was not possible for us to further identify the single fractions from the radiolabeling mixture, at least due to the missing non-radioactive reference compound (see discussion of the corresponding Re-complex earlier). These findings are in accordance to the complexation trials of ligand **4b** with (Et_4_N)_2_[Re(CO)_3_Br_3_]. In addition to the oxidized ligand **4b**, we found complexes where the phosphane moiety as monodentate ligand is bound to the Re.

**Scheme 4:**
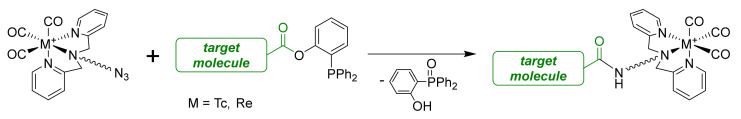
Future approach to use the indirect variant of the traceless Staudinger Ligation.

To overcome the problem of additional complexation by the phosphane moiety during the complexation step with the M(CO)_3_ core, the functionalities of labeling unit and targeting molecule have to be changed. This enables the alternative way to apply the indirect approach of the traceless Staudinger ligation (Scheme 4) for future investigations, which seems to be more promising, because organic azides are not described as ligands for the tricarbonyl core and methods to connect phosphanes to bio(macro)molecules are also known in the literature [5,37]. Moreover, the phosphanol moiety can be easily connected to pharmacological target molecules of interest with a carboxylic function.

## 3. Materials and Methods 

### 3.1. General

All reagents were purchased from commercial suppliers and were used without further purification. Analytical TLC was performed on pre-coated Silica Gel 60 F_254_ plates (Merck, Darmstadt, Germany) and results read under UV-light (λ = 254 nm). NMR spectra were recorded on an Agilent DD2-400 MHz system (OneNMR™ Probe) (Santa Clara, CA, USA) at 400 (^1^H), 101 (^13^C), 128 (^11^B), and 376 MHz (^31^P) or on an Agilent 600 MHz system (OneNMR™ Probe) at 600 (^1^H), 151 (^13^C), 192 (^11^B) and 243 MHz (^31^P). Chemical shifts are reported in ppm with tetramethylsilane (^1^H, ^13^C), BF_3_-OEt_2_ (^11^B) and H_3_PO_4_ (^31^P) as internal standard, respectively. MS spectra were obtained on a Micromass Quattro-LC spectrometer (Waters, Eschborn, Germany) using electron spray (ESI) as the ionization method. Melting points were recorded on a Galen III apparatus and are uncorrected (Cambridge Instruments/Leica, Wetzlar, Germany). Analytical HPLC was performed on a HPLC system (Perkin Elmer, Rodgau, Germany), equipped with a reverse phase column (Jupiter 4µ Proteo C18 90A (4.6 × 250 mm)), a UV-diode array detector (λ = 254 nm) and a Gabi Star scintillation radiodetector (Raytest, Straubenhardt, Germany) at a flow rate of 1 mL/min (eluent: acetonitrile/water + 0.1% TFA). The radioactive compounds were identified using analytical radio-HPLC by comparison of the retention time of the reference compound.

### 3.2. X-ray Diffraction Studies

X-ray diffraction data of **3a**, **3b**, **3c**, and **9** single-crystals were collected with a Bruker-Nonius Apex-X8 CCD diffractometer with a crystal temperature of −100 °C and those of **4a** and ***fac-*[Re(CO)_3_4a]Br** with a Bruker-Nonius Apex Kappa-II at T = −150 °C. Graphite-monochromated Mo K_α_ radiation (λ = 0.71073 Å) was used in all cases. The structures were solved by direct methods and refined against *F^2^* on all data by full-matrix least-squares using the SHELX suite of programs (version 2014/2) [38,39,40]. All non-hydrogen atoms were refined anisotropically; all hydrogen atoms bound to C atoms were placed on calculated positions and refined using riding models. The asymmetric units of **3c** and **4a** contain two of the respective title molecules. In crystals of **4a**, the O_2_C-C_6_H_4_I moiety of one of the two independent molecules is disordered with two different orientations. Furthermore, crystals of **9** contain co-crystallized CHCl_3_ molecules and those of ***fac-*[Re(CO)_3_4a]Br** co-crystallized methanol molecules. All information about the X-ray diffraction structure determinations is deposited as cif files with the CCDC data center. The data can be obtained free of charge from the Cambridge Crystallographic Data Centre: CCDC 2064011 for **3a**; CCDC 2064270 for **3b**, CCDC 2067941 for **3c**, CCDC 2071545 for **4a**, CCDC 2069525 for **9**, and CCDC 2080217 for ***fac-*[Re(CO)_3_4a]Br**.

### 3.3. Chemical Syntheses

**2-Iodophenyl 4-(chloromethyl)benzoate 3a.** KO^t^Bu (594 mg, 5.29 mmol) and 2-iodophenol (**2a**, 1.1 g, 4.81 mmol) were dissolved in anhydrous THF (10 mL). A solution of 4-(chloromethyl)benzoyl chloride (**1**, 1.0 g, 5.29 mmol) in 5 mL of anhydrous THF was added dropwise and the resulting mixture was allowed to stir overnight. Afterwards, a saturated NH_4_Cl solution (10 mL) was added, the organic layer was separated, and the aqueous layer was extracted with ethyl acetate (3 × 20 mL). The combined organic layers were dried over Na_2_SO_4_ and the solvent was removed under reduced pressure. Purification was done via column chromatography (silica gel, petroleum ether/ethyl acetate 10/1) to obtain **3a** as a colorless solid (1.8 g, 97%); mp 116 °C; R*_f_* 0.47 (petroleum ether/ethyl acetate 5/1); ^1^H NMR (400 MHz, C_6_D_6_): δ 3.92 (s, 2H, CH_2_), 6.44 (dt, ^3^*J* = 7.7 Hz, ^4^*J* = 1.6 Hz, 1H, H-4), 6.87 (t, ^3^*J* = 8.0 Hz, 1H, H-5), 6.94–6.99 (m, 3H, H-6, H_meta_), 7.53 (dd, ^3^*J* = 7.9 Hz, ^4^*J* = 1.5 Hz, 1H, H-3), 8.17 (d, ^3^*J* = 8.3 Hz, 2H, H_ortho_); ^13^C NMR (101 MHz, C_6_D_6_): δ 45.1 (CH_2_), 90.9 (C-2), 123.6 (C-6), 127.7 (C-4), 128.9 (C_meta_), 129.5 (C_ipso_), 131.0 (C_ortho_), 139.7 (C-3), 143.0 (C_para_), 152.0 (C-1), 163.7 (C=O); MS (ESI +): *m/z* = 395 [M^+^ + Na]; Anal. calcd. for: C_14_H_10_ClIO_2_ (372.59): C 45.13, H 2.71; found C 45.65, H 2.64.

**2-(Diphenylphosphano)phenyl 4-(chloromethyl)benzoate 3b.** KO^t^Bu (53 mg, 0.47 mmol) and (2-hydroxyphenyl)diphenylphosphane (**2b**, 100 mg, 0.36 mmol) were dissolved in anhydrous THF (3 mL). A solution of 4-(chloromethyl)benzoyl chloride (**1**, 88 mg, 0.47 mmol) in 1 mL of anhydrous THF was added dropwise and the resulting mixture was allowed to stir overnight. Afterwards, a saturated NH_4_Cl solution (10 mL) was added, the organic layer was separated, and the aqueous layer was extracted with ethyl acetate (3 × 20 mL). The combined organic layers were dried over Na_2_SO_4_, and the solvent was removed under reduced pressure. Purification was done via column chromatography (silica gel, petroleum ether/ethyl acetate 5/1) to obtain **3b** as a colorless solid (130 mg, 84%); mp 154 °C; R*_f_* 0.66 (petroleum ether/ethyl acetate 3/1); ^1^H NMR (400 MHz, CDCl_3_): δ 4.60 (s, 2H, CH_2_), 6.86 (dd, ^3^*J*_H,P_ = 4.5 Hz, ^3^*J* = 7.7 Hz, 1H, H-3), 7.18 (t, ^3^*J* = 7.4 Hz, 1H, H-4), 7.28–7.35 (m, 11H, H-6, H_ortho_, H_meta_, H_para_), 7.37 (d, ^3^*J* = 8.3 Hz, 2H, H_meta’_), 7.43 (t, ^3^*J* = 7.7 Hz, 1H, H-5), 7.82 (d, ^3^*J* = 8.3 Hz, 2H, H_ortho’_); ^13^C NMR (101 MHz, CDCl_3_): δ 45.5 (CH_2_), 122.6 (d, ^3^*J*_C.P_ = 1.6 Hz, C-6), 126.4 (C-4), 128.5 (C_meta’_), 128.7 (d, ^3^*J*_C,P_ = 7.3 Hz, C_meta_), 129.2 (C_para_), 130.0 (C_ortho’_), 130.1 (d, ^1^*J*_C,P_ = 106.7, C_ipso_), 130.7 (C_ortho_), 133.7 (d, ^2^*J*_C,P_ = 2.0 Hz, C-5), 134.2 (d, ^2^*J*_C,P_ = 20.6 Hz, C_ortho_), 135.5 (d, ^4^*J*_C,P_ = 10.2, C-3), 142.8 (C_para’_), 152.8 (d, ^2^*J*_C,P_ = 16.3 Hz, C-1), 163.9 (C=O); ^31^P NMR (162 MHz, CDCl_3_): δ −14.4 ppm; MS (ESI+): *m/z* = 431 (28) [M^+^ + H], 453 (12) [M^+^ + Na]; Anal. calcd. for: C_26_H_20_ClO_2_P (430.86): C 72.48, H 4.68; found C 72.70, H 4.85.

**2-(Diphenylphosphano)phenyl 4-(chloromethyl)benzoate borane adduct 3c.** KO^t^Bu (254 mg, 2.26 mmol) and (2-hydroxyphenyl)diphenylphosphane borane (**2c**, 440 mg, 1.51 mmol) were dissolved in anhydrous THF (5 mL). A solution of 4-(chloromethyl)benzoyl chloride (427 mg, 2.26 mmol) in 2 mL of anhydrous THF was added dropwise and the resulting mixture was allowed to stir overnight. Afterwards, a saturated NH_4_Cl solution (10 mL) was added, the organic layer was separated, and the aqueous layer was extracted with ethyl acetate (3 × 20 mL). The combined organic layers were dried over Na_2_SO_4_, and the solvent was removed under reduced pressure. Purification was done via column chromatography (silica gel, petroleum ether/ethyl acetate 5/1) to obtain **3c** as a colorless solid (548 mg, 82%); mp 128 °C; R*_f_* 0.34 (petroleum ether/ethyl acetate 5/1); ^1^H NMR (400 MHz, C_6_D_6_): δ 1.67–2.63 (m, 3H, BH_3_), 3.88 (s, 2H, CH_2_), 6.74 (t, ^3^*J* = 7.5 Hz, 1H, H-4), 6.81 (d, ^3^*J* = 8.3 Hz, 2H, H_meta’_), 6.89–6.93 (m, 6H, H_ortho_, H_meta_), 7.03 (t, ^3^*J* = 7.8 Hz, 1H, H-5), 7.17 (ddd, ^4^*J*_H,P_ = 3.9 Hz, H-6), 7.29 (ddd, ^4^*J* = 1.6 Hz, ^3^*J* = 7.3 Hz, ^3^*J*_H,P_ = 11.6 Hz, H-3), 7.69–7.76 (m, 4H, H_para_), 7.80 (d, ^3^*J* = 8.3 Hz, 2H, H_ortho’_); ^13^C NMR (101 MHz, C_6_D_6_): δ 45.1 (CH_2_), 124.7 (d, *J* = 4.9 Hz, C-6), 126.0 (d, *J* = 9.1 Hz, C-4), 128.4 (C_meta’_), 128.9 (d, ^3^*J* = 10.2 Hz, C_meta_), 129.4 (C-5), 130.9 (C_ortho’_), 131.2 (^4^*J*_C,P_ = 2.4 Hz, C_para_), 132.6 (d, *J* = 2.0 Hz, C-3), 133.5 (d, ^2^*J* = 9.8 Hz, C_ortho_), 134.9 (d, ^1^*J*_C,P_ = 7.3 Hz, C_ipso_), 143.0 (C_para’_), 153.2 (d, ^1^*J*_C,P_ = 3.2 Hz, C-1), 163.3 (C=O); ^31^P NMR (162 MHz, C_6_D_6_): δ 20.9 ppm; ^11^B NMR (MHz, C_6_D_6_): δ −36.6 ppm; MS (ESI+): *m/z* = 467 (20) [M^+^ + Na]; Anal. calcd. for: C_26_H_23_BClO_2_P (444.70): C 70.22, H 5.21; found C 70.41, H 5.31.

**2-Iodophenyl 4-((bis(pyridin-2-ylmethyl)amino)methyl)benzoate 4a**. Compound **3a** (500 mg, 1.34 mmol) was dissolved in acetone (6 mL), NaI (221 mg, 1.47 mmol) was added and the mixture was stirred for 1 h at rt. Afterwards, 2,2′-dipicolylamine (335 mg, 1.68 mmol) was added and the resulting mixture was stirred overnight at rt. Thereafter, the solvent was removed, saturated hydrogen carbonate solution (20 mL) was added, and the aqueous layer was extracted with ethyl acetate (3 × 20 mL). The combined organic layers were dried over Na_2_SO_4_ and the solvent was removed under reduced pressure. Purification was performed via column chromatography (silica gel, ethyl acetate → ethyl acetate/EtOH 10/1) to obtain **4a** as a colorless solid (620 mg, 86%); mp 90 °C; R*_f_* 0.41 (chloroform/methanol 9/1); ^1^H NMR (400 MHz, C_6_D_6_): δ 3.60 (s, 2H, CH_2_N), 3.86 (s, 4H, CH_2_Pyr), 6.44 (dt, ^3^*J* = 7.7 Hz, ^4^*J* = 1.4 Hz, 1H, H-4), 6.65 (dd, ^3^*J* = 4.9 Hz, ^3^*J* = 7.4 Hz, 2H, Pyr), 6.87 (dt, ^3^*J* = 8.0 Hz, ^4^*J* = 1.4 Hz, 1H, H-5), 7.02 (dd, ^3^*J* = 8.0 Hz, ^4^*J* = 1.4 Hz, 1H, H-6), 7.13 (dd, ^3^*J* = 7.6 Hz, ^4^*J* = 2.0 Hz, 2H, Pyr), 7.37 (d, ^3^*J* = 7.7 Hz, 2H, Pyr), 7.41 (d, ^3^*J* = 8.2 Hz, 2H, H_meta_), 7.54 (dd, ^3^*J* = 7.8 Hz, ^4^*J* = 1.4 Hz, 1H, H-3), 8.31 (d, ^3^*J* = 8.2 Hz, 2H, H_ortho_), 8.48 (d, ^3^*J* = 4.9 Hz, 2H, Pyr); ^13^C NMR (101 MHz, C_6_D_6_): δ 58.3, 60.2 (CH_2_), 91.1 (C-2), 122.0 (C-5-Pyr), 122.9 (C-3-Pyr), 123.7 (C-6), 127.5 (C-4), 128.6 (C_ipso_), 129.3 (C_meta_), 129.4 (C-5), 130.9 (C_ortho_), 136.0 (C-4-Pyr), 139.6 (C-3), 146.5 (C_para_), 149.5 (C-6-Pyr), 152.1 (C-1), 160.0 (C-2-Pyr), 164.2 (C=O); MS (ESI+): *m/z* = 536 (100) [M^+^ + H]; Anal. calcd. for: C_26_H_22_IN_3_O_2_ (535.38): C 58.33, H 4.14; found C 58.36, H 4.11.

**2-(Diphenylphosphino)phenyl 4-((bis(pyridin-2-ylmethyl)amino)methyl)benzoate 4b**. Under argon, compound **3b** (250 mg, 0.58 mmol) was dissolved in anhydrous THF (5 mL), NaI in catalytic amounts and 2,2′-dipicolylamine (193 mg, 0.97 mmol) was added and the resulting mixture was stirred at 60 °C for 5 h. Thereafter, the solvent was removed, saturated hydrogen carbonate solution (20 mL) was added, and the aqueous layer was extracted with ethyl acetate (3 × 20 mL). The combined organic layers were dried over Na_2_SO_4_, and the solvent was removed under reduced pressure. Purification was done via column chromatography (silica gel, ethyl acetate) to obtain compound **4b** as a pale yellow solid (224 mg, 65%); mp 77 °C; R*_f_* 0.45 (chloroform/methanol 9/1); ^1^H NMR (400 MHz, CDCl_3_): δ 3.73 (s, 2H, CH_2_N); 3.81 (s, 4H, CH_2_Pyr), 6.85 (dd, ^4^*J* = 4.2 Hz, ^3^*J* = 7.4 Hz, 1H, H-4), 7.13–7.18 (m, 3H, H-1, Pyr), 7.25–7.34 (m, 11H, H_ortho_, H_meta_, H_para_, H-5), 7.38–7.43 (m, 3H, H_meta’_, H-2), 7.55 (d, ^3^*J* = 7.9 Hz, 2H, Pyr), 7.68 (dt, ^4^*J* = 1.7 Hz, ^3^*J* = 7.7 Hz, 2H, Pyr), 7.80 (d, ^3^*J* = 8.3 Hz, 2H, H_ortho’_), 8.53 (d, ^3^*J* = 4.4 Hz, 2H, Pyr); ^13^C NMR (101 MHz, CDCl_3_): δ 58.4 (NCH_2_Ar), 60.2 (NCH_2_Py), 122.2 (C-Py-5), 122.7 (C-6), 123.0 (C-Py-3), 126.2 (C-5), 128.1 (C-i’), 128.7 (d, ^3^*J*_C,P_ = 7.4 Hz, C-m, C-m’), 129.1 (C-p), 123.0 (C-2), 130.3 (C-o’), 130.8 (d, ^1^*J*_C,P_ = 15.0 Hz, C-i), 133.6 (C-4), 134.2 (d, ^2^*J* = 20.7 Hz, C-o), 135.6 (d, ^2^*J*_C,P_ = 10.2 Hz, C-3), 136.6 (C-Py-4), 145.3 (C-p’), 149.2 (C-Py-6), 152.9 (d, ^2^*J*_C,P_ = 17.0 Hz, C-1), 159.4 (C-Py-2); 164.2 (C=O); ^31^P NMR (162 MHz, CDCl_3_): δ −14.8; MS (ESI+): *m/z* (%) = 594 (100) [M^+^ + H], 616 (60) [M^+^ + Na]; Anal. calcd. for: C_38_H_32_N_3_O_2_P (593.65): C 76.88, H 5.43; found C 76.91, H 5.51.

***fac-*[Re(CO)_3_4a]Br.** Compound **4a** (100 mg, 0.14 mmol) and (NH_4_)_2_[Re(CO)_3_Br_3_] (143 mg, 0.14 mmol) were dissolved in methanol (5 mL) and the resulting mixture was stirred at 60 °C for 6 h. Afterwards, the solvent was removed and the crude product was dissolved in chloroform (15 mL), the organic phase was washed with water (2 × 10 mL) and dried over Na_2_SO_4_. Afterwards, petroleum ether was added to precipitate the complex. Compound **5a** was obtained as a colorless solid (157 mg, 95%); mp 215°C; ^1^H NMR (400 MHz, CDCl_3_): δ 4.73 (d, ^2^*J* = 16.3 Hz, 1H, CH_2_Pyr), 4.96 (s, 2H, CH_2_N), 5.84 (d, ^2^*J* = 16.3 Hz, 1H, CH_2_Pyr), 7.02 (dt, ^3^*J* = 4.9 Hz, ^3^*J* = 7.5 Hz, 1H, H-4), 7.19 (t, ^3^*J* = 6.5 Hz, 2H, H-5-Pyr), 7.24–7.28 (m, 1H, H-6), 7.41 (dt, ^3^*J* = 7.5 Hz, ^4^*J* = 2.0 Hz, 1H, H-5), 7.79–7.88 (m, 3H, H-3, H-4-Pyr), 7.93 (d, ^3^*J* = 8.1 Hz, 2H, H_meta_), 8.01 (d, ^3^*J* = 8.0 Hz, 2H, H-3-Pyr), 8.37 (d, ^3^*J* = 8.1 Hz, 2H, H_ortho_), 8.63 (d, ^3^*J* = 5.6 Hz, 2H, H-6-Pyr); ^13^C NMR (101 MHz, CDCl_3_): δ 68.1 (CH_2_Pyr), 72.0 (CH_2_N), 90.5 (C-2), 123.3 (C-6), 125.5, 125.6 (C-3Pyr/C-5Pyr), 127.9 (C-4), 129.6 (C-5), 130.9 (C_ipso_), 131.4 (C_ortho_), 133.3 (C_meta_), 137.6 (C_para_), 139.6 (C-3), 140.6 (C-4_Pyr_), 150.8 (C-6Pyr), 151.3 (C-1), 160.4 (C-2_Pyr_), 163.7 (C=O), 195.2, 195.8 (3 × C≡O). MS (ESI+): *m/z* = 804 (65) [M^+^ − Br, ^185^Re], 806 (100) [M^+^ − Br, ^187^Re]; Anal. calcd. for: C_29_H_22_BrIN_3_O_5_Re (884.93): C 39.33, H 2.84, N 4.75; found C 39.36, H 2.81, N 4.77. Analytical HPLC (254 nm): t_R_ = 19.8 min.

**2-(Diphenylphosphoryl)phenylbenzoate 7.** Compound **6** [24] (210 mg, 0.44 mmol) was dissolved in DMF (4 mL), NaN_3_ (113 mg, 1.75 mmol) and water (0.5 mL) were added, and the solution was stirred at 80 °C for 4 h. Afterwards, the solvent was removed and the crude product was dissolved in chloroform (20 mL), the aqueous phase was washed with water (3 × 15 mL), dried over Na_2_SO_4_ and the solvent was removed. Purification was performed by column chromatography (petroleum ether/ethyl acetate, 1/1) to yield compound **6** (161 mg, 90%) as a colorless oil. R*_f_* 0.29 (petroleum ether/ethyl acetate, 1/1). ^1^H NMR (400 MHz, CDCl_3_): δ 6.78–6.84 (m, 1H, H-5), 6.95 (ddd, ^3^*J* = 14.7 Hz, ^3^*J* = 7.8 Hz, ^4^*J* = 1.6 Hz, 1H, H-3), 7.07 (ddd, ^3^*J* = 8.4 Hz, ^3^*J* = 5.0, ^4^*J* = 0.7 Hz, 1H, H-6), 7.39–7.63 (m, 10H, H-m’, H-m, H-p, H-p’, H-4), 7.81–7.88 (m, 4H, H-o), 8.15-8.19 (m, 2H, H-o’) ppm; ^13^C NMR (101 MHz, CDCl_3_): δ = 107.7 (d, *J*_C,P_ = 110.4 Hz), 119.3 (d, *J*_C,P_ = 13.3 Hz), 119.6 (d, *J*_C,P_ = 8.8 Hz), 126.5, 127.4, 128.2 (CH_Ar_), 129.0 (d, *J*_C,P_ =12.5 Hz, CH_Ar_), 129.3 (d, *J*_C,P_ = 2.2 Hz, CH_Ar_), 131.6, 132.9 (d, *J*_C,P_ 2.9 = Hz), 133.3 (d, *J*_C,P_ = 10.7 Hz, CH_Ar_), 133.4 (d, *J*_C,P_ = 11.3 Hz), 135.0 (d, *J*_C,P_ = 2.5 Hz), 137.0 (d, *J*_C,P_ = 2.6 Hz), 164.2 (d, *J*_C,P_ = 17.3 Hz, C=O), 176.1 (d, *J*_C,P_ = 8.6 Hz) ppm; ^31^P NMR (162 MHz, CDCl_3_): δ 29.7 ppm; MS (ESI+): *m/z* (%) = 399 [M^+^ + H], 421 [M^+^ + Na]; Anal. calcd. for: C_25_H_19_O_3_P (398.40): C 75.37, H 4.81; found C 75.28, H 4.85.

***fac-*[Re(CO)_3_(5)_2_Br] 8**. Compound **6** (102 mg, 0.36 mmol) and (Et_4_N)_2_[Re(CO)_3_Br_3_] (274 mg, 0.36 mmol) were dissolved in methanol (8 mL) and stirred for 7 h at 60 °C. Afterwards, the solvent was removed and the crude product purified using RP column chromatography (water/acetonitrile 10:1 → 1:1). After lyophilization, compound **8** (23 mg, 19%) was obtained as colorless solid. R*_f_* 0.54 (RP-18, methanol). ^1^H NMR (400 MHz, C_6_D_6_): δ 6.78 (t, 1H, 3*J* = 7.5 Hz, H-p’), 6.89–7.09 (m, 10H, H-m, H-m’, H-p, H-4, H-6), 7.17–7.22 (m, 1H, H-5), 7.52 (dd, 1H, ^3^*J*_H,P_ = 7.4 Hz, ^3^*J* = 12.5 Hz, H-3), 7.75–7.81 (m, 4H, H-o), 8.01 (d, 2H, ^3^*J* = 7.4 Hz, H-o’) ppm; ^13^C NMR (101 MHz, C_6_D_6_): δ 124.5 (d, ^3^*J*_C,P_ = 5.9 Hz, C-6), 125.6 (d, ^3^*J*_C,P_ = 11.3 Hz, C-4), 128.4 (c-m), 128.6 (C-p), 129.4 (C-5), 130.8 (C-o’), 131.5 (d, ^1^*J*_C,P_ = 2.6 Hz, C-2), 132.2 (d, ^1^*J*_C,P_ = 9.8 Hz, c-o), 133.3 (d, ^2^*J*_C,P_ = 4.0 Hz, C-3), 134.6 (d, ^1^*J*_C,P_ = 8.3 Hz, C-i), 153.7 (C-1), 163.9 (C=O) ppm (missing signals are under the solvent signal); ^31^P NMR (162 MHz, C_6_D_6_): δ 23.1 ppm; IR (ATR): ν = 3536 (OH), 3403 (OH), 2029 (C≡O), 1909 (C≡O) cm^−1^; MS (ESI+): *m/z* 1118 [M^+^ + H, ^81^Br], 1116 [M^+^ + H, ^79^Br].

***fac-*Acetonitrile-tris-carbonyl-(2-(diphenylposphino)phenolato)-rhenium(I) 9**. Under argon, 2-(diphenylphosphano)phenol (**2b**, 99 mg, 0.36 mmol) and (Et_4_N)_2_[Re(CO)_3_Br_3_] (274 mg, 0.36 mmol) were dissolved in methanol (8 mL) and stirred for 7 h at 60 °C. Afterwards, the solvent was removed and the crude product purified using RP column chromatography (water/acetonitrile 10:1 → 1:1). After lyophilization, compound **9** (79 mg, 38%) was obtained as a colorless solid. R*_f_* 0.62 (RP-18, methanol); mp 108 °C; ^1^H NMR (400 MHz, C_6_D_6_): δ 6.53–6.60 (m, 1H, H-4), 6.86–7.04 (m, 6H, H-3, H-6, H-m), 7.18–7.23 (m, 1H, H-5), 7.28–7.38 (m, 2H, H-p), 7.59–7-69 (m, 2H, H-o), 7.76 (dd, ^3^*J* = 7.8 Hz, ^3^*J* = 11.8 Hz, 2H, H-o) ppm; ^13^C NMR (101 MHz, C_6_D_6_): δ 113.0 (d, ^1^*J*_C,P_ = 53.6 Hz, C-2), 115.7 (d, ^3^*J*_C,P_ = 5.8 Hz, C-6), 122.3 (d, ^3^*J*_C,P_ = 7.6 Hz, C-4), 129.0 (d, ^3^*J*_C,P_ = 10.7 Hz, C-m), 129.0 (d, ^3^*J*_C,P_ = 9.8 Hz, C-m), 459.8 (C-p), 123.6 (C-p), 132.8 (d, ^2^*J*_C,P_ = 11.6 Hz, C-o), 133.4 (d, ^2^*J*_C,P_ = 10.7 Hz, C-o), 133.7 (C-5), 134.0 (C-3), 180.7 (d, ^2^*J*_C,P_ = 25.4 Hz, C-1) ppm; ^31^P NMR (202 MHz, C_6_D_6_): δ = 32.9 ppm; IR (ATR): ν = 3399 (OH), 2020 (C≡O), 1923 (C≡O), 1884 (C≡O) cm^−1^; MS (ESI+): *m/z* (%) = 549 (100) [M^+^-CH_3_CN]; Anal. calcd. for: C_23_H_17_NO_4_PRe (588.57): C 46.94, H 2.91, N 2.38; found C 46.64, H 2.66, N 2.01.

### 3.4. Radiolabeling Procedure

The preparation of boranocarbonate and the tricarbonyl kit (content: 17 mg Na-tartrate, 3.5 mg Na_2_B_4_O_7_, 3.2 mg Na_2_CO_3_, 8.1 mg K_2_[CO_2_BH_3_]) was described earlier; see ref. [41]. ^99m^Tc was used as Na[^99m^Tc]TcO_4_ generator eluate obtained by elution from a ^99^Mo/^99m^Tc generator (Polatom, Otwok, Poland) using a 0.9% NaCl solution. [^99m^Tc]TcO_4_ (1 mL, 500 MBq) was added to the tricarbonyl kit. Afterwards, the solution was heated for 30 min at 100 °C, cooled down to rt for 15 min, and was ready to use without further purification. Next, the pH of the *fac*-[^99m^Tc(CO)_3_(H_2_O)_3_]^+^ (analytical radio HPLC: t_R_ = 14.1 min) complex solution was adjusted to 6.2 by adding MES buffer (500 µL). Ligand **4a** or **4b** (200 µg) was dissolved in 200 µL of ethanol and added. The resulting reaction mixture was stirred at 100 °C for 30 min. Radio-HPLC showed 78% product formation. After cooling to rt and filtration, the solution was added to a RP-18 cartridge (LiChrolut RP-18 (40–63 µm), 500 mg) for purification. The cartridge was washed with water (3 × 2 mL) and eluted with ethanol (2 mL) to yield the final radiotracer. 

The resulting radiotracer was analyzed via HPLC using a Jupiter 4µ Proteo C18 90A (4.6 × 250 mm) column (phenomenex, Aschaffenburg, Germany). Eluents were purified water + 0.1% TFA (solvent A) and acetonitrile + 0.1% TFA (solvent B) with a gradient of 95% (A) 0–3 min, 95% → 5% (A) 3–20 min, 5% (A) 20–25 min, 5 → 95% (A) 25–28 min, 95% (A) 28–45 min (flow rate: 1 ml/min). Analytical radio HPLC for ***fac-*[[^99m^Tc]Tc(CO)_3_4a]^+^**: t_R_ = 20.2 min.

## 4. Conclusions

A proof-of-concept study was carried out to use the direct variant of the traceless Staudinger ligation for radiolabeling purposes with a ^99m^Tc-tricarbonyl core. For this purpose, phosphane **4b** with benzoate moiety was prepared and functionalized by introducing a dipicolylamine residue as tridentate ligand. Reactions with (Et_4_N)_2_[Re(CO)_3_Br_3_] to prepare the non-radioactive reference led to a mixture of products, including the oxidized **4b** and different Re-complexes, which could not be identified completely. The same situation was found for the radiolabeling of **4b** with *fac*-[^99m^Tc(CO)_3_(H_2_O)_3_]^+^. Due to the high number of by-products during complexation, which were mandatory to separate, and the resultant low yields, it must be stated that this variant of the Staudinger ligation is not sufficient for radiolabeling purposes. Future work will entail concentrating on the change of the functionalities to prepare target molecules containing the phosphanol residue and azide-functionalized DPA-containing radiolabeling building blocks for chelation of the ^99m^Tc-tricarbonyl core.

## Data Availability

All data are included in this paper.

## Supplementary Materials

The following are available online, NMR spectra of compounds, radiolabeling procedure and radio HPLC chromatograms.

## Sample Availability

Samples of the compounds are not available from the authors.

## References

[1] Köhn M., Breinbauer R. (2004). Die Staudinger-Ligation—Ein Geschenk für die Chemische Biologie. Angew. Chem..

[2] Schilling C.I., Jung N., Biskup M., Schäpers U., Bräse S. (2011). Bioconjugation via azide—Staudinger ligation: An overview. Chem. Soc. Rev..

[3] Van Berkel S.S., van Eldijk M.B., van Hest J.C.M. (2011). Staudinger-Ligation als Methode zur Biokonjugation. Angew. Chem..

[4] Wang Z.-P.A., Tian C.-L., Zheng J.-S. (2015). The recent developments and applications of the traceless - Staudinger reaction in chemical biology study. RSC Adv..

[5] Bednarek C., Wehl I., Jung N., Schepers U., Braäse S. (2020). The Staudinger Ligation. Chem. Rev..

[6] Pretze M., Pietzsch D., Mamat C. (2013). Recent Trends in Bioorthogonal Click-Radiolabeling Reactions Using Fluorine-18. Molecules.

[7] Mamat C., Gott M., Steinbach J. (2018). Recent progress using the Staudinger Ligation for radiolableing purposes. J. Label. Compd. Radiopharm..

[8] Wodtke R., König J., Pigorsch A., Köckerling M., Mamat C. (2015). Evaluation of novel fluorescence probes for conjugation purposes using the traceless Staudinger ligation. Dyes Pigm..

[9] Pretze M., Wuest F., Peppel T., Köckerling M., Mamat C. (2010). The traceless Staudinger ligation with fluorine - 18: A novel and versatile labeling technique for the synthesis of PET - radiotracers. Tetrahedron Lett..

[10] Alberto R., Ortner K., Wheatley N., Schibli R., Schubiger P.A. (2001). Synthesis and Properties of Boranocarbonate: A Convenient in Situ CO Source for the Aqueous Preparation of [^99m^Tc(OH_2_)_3_(CO)_3_]^+^. J. Am. Chem. Soc..

[11] Alberto R., Schibli R., Egli A., Schubiger P.A. (1998). A Novel Organometallic Aqua Complex of Technetium for the Labeling of Biomolecules: Synthesis of [^99m^Tc(OH_2_)_3_(CO)_3_]^+^ from [^99m^TcO_4_]^−^ in Aqueous Solution and Its Reaction with a Bifunctional Ligand. J. Am. Chem. Soc..

[12] Alberto R., Schibli R., Waibel R., Abram U., Schubiger P.A. (1999). Basic aqueous chemistry of [M(OH_2_)_3_(CO)_3_]^+^ (M=Re, Tc) directed towards radiopharmaceutical application. Coord. Chem. Rev..

[13] Porchia M., Bolzati C., Refosco F., Vittadini A. (2006). The preparation of substitution-inert ^99^Tc metal-fragments: Promising candidates for the design of new ^99m^Tc radiopharmaceuticals. Coord. Chem. Rev..

[14] Mindt T.L., Struthers H., Brans L., Anguelov T., Schweinsberg C., Maes V., Tourwé D., Schibli R. (2006). “Click to Chelate”: Synthesis and Installation of Metal Chelates into Biomolecules in a Single Step. J. Am. Chem. Soc..

[15] Mindt T.L., Müller mC., Melis M., de Jong M., Schibli R. (2008). “Click-to-Chelate”: In Vitro and In Vivo Comparison of a ^99m^Tc(CO)_3_-Labeled N(τ)-Histidine Folate Derivative with Its Isostructural, Clicked 1,2,3-Triazole Analogue. Bioconjug. Chem..

[16] Kluba C.A., Mindt T.L. (2013). Click-to-Chelate: Development of Technetium and Rhenium-Tricarbonyl Labeled Radiopharmaceuticals. Molecules.

[17] Mamat C., Ramenda T., Wuest F.R. (2009). Recent Applications of Click Chemistry for the Synthesis of Radiotracers for Molecular Imaging. Mini-Rev. Org. Chem..

[18] Choi J.Y., Lee B.C. (2015). Click Reaction: An Applicable Radiolabeling Method for Molecular Imaging. Nucl. Med. Mol. Imaging.

[19] Meyer J.P., Adumeau P., Lewis J.S., Zeglis B.M. (2016). Click Chemistry and Radiochemistry: The First 10 Years. Bioconjug. Chem..

[20] James S., Maresca K.P., Allis D.G., Valliant J.F., Eckelman W., Babich J.W., Zubieta J. (2006). Extension of the Single Amino Acid Chelate Concept (SAAC) to Bifunctional Biotin Analogues for Complexation of the M(CO)_3_^+1^ Core (M = Tc and Re): Syntheses, Characterization, Biotinidase Stability, and Avidin Binding. Bioconjug. Chem..

[21] Maresca K.P., Hillier S.M., Femia F.J., Zimmerman C.N., Levadala M.K., Banerjee S.R., Hicks J., Sundararajan C., Valliant J., Zubieta J. (2009). Comprehensive Radiolabeling, Stability, and Tissue Distribution Studies of Technetium-99m Single Amino Acid Chelates (SAAC). Bioconjug. Chem..

[22] Hayes T.R., Lyon P.A., Silva-Lopez E., Twamley B., Benny P.D. (2013). Photo-initiated Thiol-ene Click Reactions as a Potential Strategy for Incorporation of [M^I^(CO)_3_]^+^ (M = Re, ^99m^Tc) Complexes. Inorg. Chem..

[23] Banerjee S.R., Levadala M.K., Lazarova N., Wei L., Valliant J.F., Stephenson K.A., Babich J.W., Maresca K.P., Zubieta J. (2002). Bifunctional Single Amino Acid Chelates for Labeling of Biomolecules with the {Tc(CO)_3_}^+^ and {Re(CO)_3_}^+^ Cores. Crystal and Molecular Structures of [ReBr(CO)_3_(H_2_NCH_2_C_5_H_4_N)], [Re(CO)_3_{(C_5_H_4_NCH_2_)_2_NH}]Br, [Re(CO)_3_{(C_5_H_4_NCH_2_)_2_NCH_2_CO_2_H}]Br, [Re(CO)_3_{X(Y)NCH_2_CO_2_CH_2_CH_3_}]Br(X = Y = 2-pyridylmethyl; X = 2-pyridylmethyl, Y = 2-(1-methylimidazolyl)methyl; X = Y = 2-(1-methylimidazolyl)methyl), [ReBr(CO)_3_{(C_5_H_4_NCH_2_)NH(CH_2_C_4_H_3_S)}], and [Re(CO)_3_{(C_5_H_4_NCH_2_)N(CH_2_C_4_H_3_S)(CH_2_CO_2_)}]. Inorg. Chem..

[24] Mamat C., Flemming A., Köckerling M., Steinbach J., Wuest F.R. (2009). Synthesis of benzoate - functionalized phosphanes as novel building blocks for the traceless Staudinger ligation. Synthesis.

[25] Mamat C., Köckerling M. (2015). Preparation of 4-halobenzoate-containing phosphane-based building blocks for labeling reactions using the traceless Staudinger ligation. Synthesis.

[26] Alberto R., Egli A., Abram U., Hegetschweiler K., Gramlich V., Schubiger P.A. (1994). Synthesis and reactivity of [NEt_4_]_2_[ReBr_3_(CO)_3_]. Formation and structural characterization of the clusters [NEt_4_][Re_3_(µ_3_-OH)(µ-OH)_3_(CO)_9_] and [NEt_4_][Re_2_(µ-OH)_3_(CO)_6_] by alkaline titration. J. Chem. Soc., Dalton Trans..

[27] Braband H., Abram U. (2004). Tricarbonyl complexes of rhenium(I) and technetium(I) thiourea complexes. J. Organomet. Chem..

[28] Abram U., Abram S., Alberto R., Schibli R. (1996). Ligand exchange reactions starting from [Re(CO)_3_Br_3_]^2−^. Synthesis, characterization and structures of rhenium(I) tricarbonyl complexes with thiourea and thiourea derivatives. Inorg. Chim. Acta.

[29] Lepareur N., Lacœuille F., Bouvry C., Hindré F., Garcion E., Chérel M., Noiret N., Garin E., Knapp F.F.R. (2019). Rhenium-188 Labeled Radiopharmaceuticals: Current Clinical Applications in Oncology and Promising Perspectives. Front. Med..

[30] Dilworth J.R., Parrott S.J. (1998). The biomedical chemistry of technetium and rhenium. Chem. Soc. Rev..

[31] Alberto R., Alessio E. (2011). Metal-Based Radiopharmaceuticals. Bioinorganic Medicinal Chemistry.

[32] Gomes Marin J.F., Nunes R.F., Coutinho A.M., Zaniboni E.C., Costa L.B., Barbosa F.G., Queiroz M.A., Cerri G.G., Buchpiguel C.A. (2020). Theranostics in Nuclear Medicine: Emerging and Re-emerging Integrated Imaging and Therapies in the Era of Precision Oncology. Radiographics.

[33] Yordanova A., Eppard E., Kürpig S., Bundschuh R.A., Schönberger S., Gonzalez-Carmona M., Feldmann G., Ahmadzadehfar H., Essler M. (2017). Theranostics in nuclear medicine practice. OncoTargets Ther..

[34] Horn E., Onai S. (2001). Crystal structure of *cis*-bis(triphenylphosphine)-*fac*-(tricarbonyl)-rhenium(I) bromide, Re(CO)_3_(C_18_H_15_P)_2_Br. Z. Krist. NCS.

[35] Shegani A., Triantis C., Nock B.A., Maina T., Kiritsis C., Psycharis V., Raptopoulou C., Pirmettis I., Tisato F., Papadopoulos M.S. (2017). Rhenium(I) Tricarbonyl Complexes with (2-Hydroxyphenyl)diphenylphosphine as PO Bidentate Ligand. Inorg. Chem..

[36] Gao F., Sihver W., Bergmann R., Belter B., Bolzati C., Salvarese N., Steinbach J., Pietzsch J., Pietzsch H.-J. (2018). Synthesis, Characterization, and Initial Biological Evaluation of [^99m^Tc]Tc-Tricarbonyl-labeled DPA-α-MSH Peptide Derivatives for Potential Melanoma Imaging. ChemMedChem.

[37] Grandjean C., Boutonnier A., Guerreiro C., Fournier J.-M., Mulard L.A. (2005). On the Preparation of Carbohydrate−Protein Conjugates Using the Traceless Staudinger Ligation. J. Org. Chem..

[38] Sheldrick G.M. (2015). Crystal structure refinement with SHELXL. Acta Cryst..

[39] Sheldrick G.M. (2008). A short history of SHELX. Acta Cryst..

[40] Sheldrick G.M. (2015). SHELXT—Integrated space-group and crystal-structure determination. Acta Cryst..

[41] Reissig F., Mamat C., Steinbach J., Pietzsch H.-J., Freudenberg R., Navarro-Retamal C., Caballero J., Kotzerke J., Wunderlich G. (2016). Direct and Auger electron-induced, single- and double-strand breaks on plasmid DNA caused by ^99m^Tc-labeled pyrene derivatives and the effect of bonding distance. PLoS ONE.

