# Supplementary material for: Strategic Evaluation of the Traceless Staudinger Ligation for Radiolabeling with the Tricarbonyl Core
Source: Molecules. 2021 Nov 1;26(21):6629. doi: 10.3390/molecules26216629 (PMC8587073; doi:10.3390/molecules26216629)

Supporting Information

Strategic Evaluation of the Traceless Staudinger Ligation for Radiolabeling with the Tricarbonyl Core

Constantin Mamat [1,2,*], Christian Jentschel [1], Martin Köckerling [3] and Jörg Steinbach [1,2]

[1] Helmholtz-Zentrum Dresden-Rossendorf, Institut für Radiopharmazeutische Krebsforschung, Bautzner Landstraße 400, D-01328 Dresden, Germany; c.jentschel@hzdr.de (C.J.), steinbach-joerg@web.de (J.S.)
[2] Fakultät Chemie und Lebensmittelchemie, Technische Universität Dresden, D-01062 Dresden, Germany
[3] Institut für Chemie, Anorganische Festkörperchemie, Universität Rostock, Albert-Einstein-Straße 3a, D-18059 Rostock, Germany; martin.koeckerling@uni-rostock.de
* Correspondence: c.mamat@hzdr.de

Table of Contents

NMR spectra of compounds SI-2

HPLC chromatogram of ligands **4a** and **4b** SI-11

HPLC chromatogram of rhenium complex ***fac*-[Re(CO)$_3$]Br** SI-12

Content of the tricarbonyl kit, HPLC conditions and radiolabeling SI-13

Radiolabeling procedure of ligands **4a** and **4b** SI-14

Radio HPLC chromatograms of ***fac*-[[^{99m}Tc]Tc(CO)$_3$4a]$^+$** SI-15

Radio HPLC chromatograms of *fac*-**[[^{99m}Tc]Tc(CO)$_3$4b]$^+$** SI-16

NMR spectra of compounds

Compound **3a**

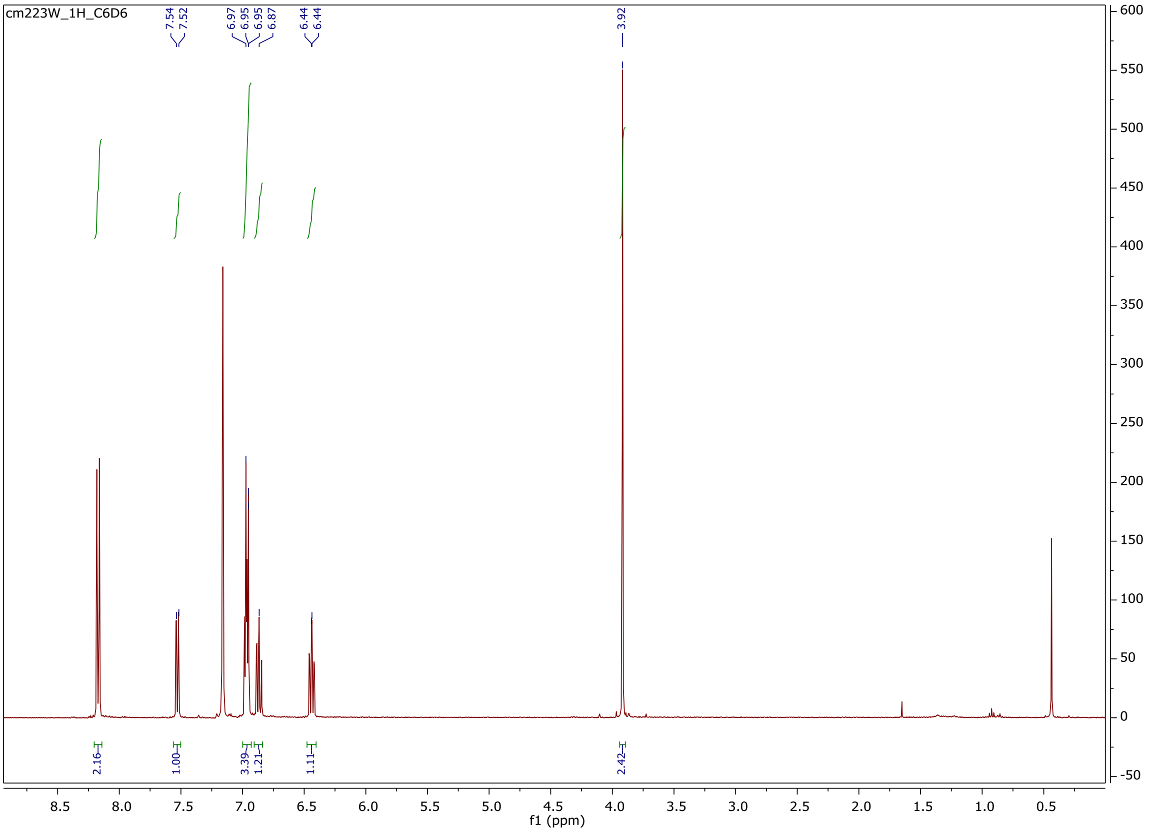

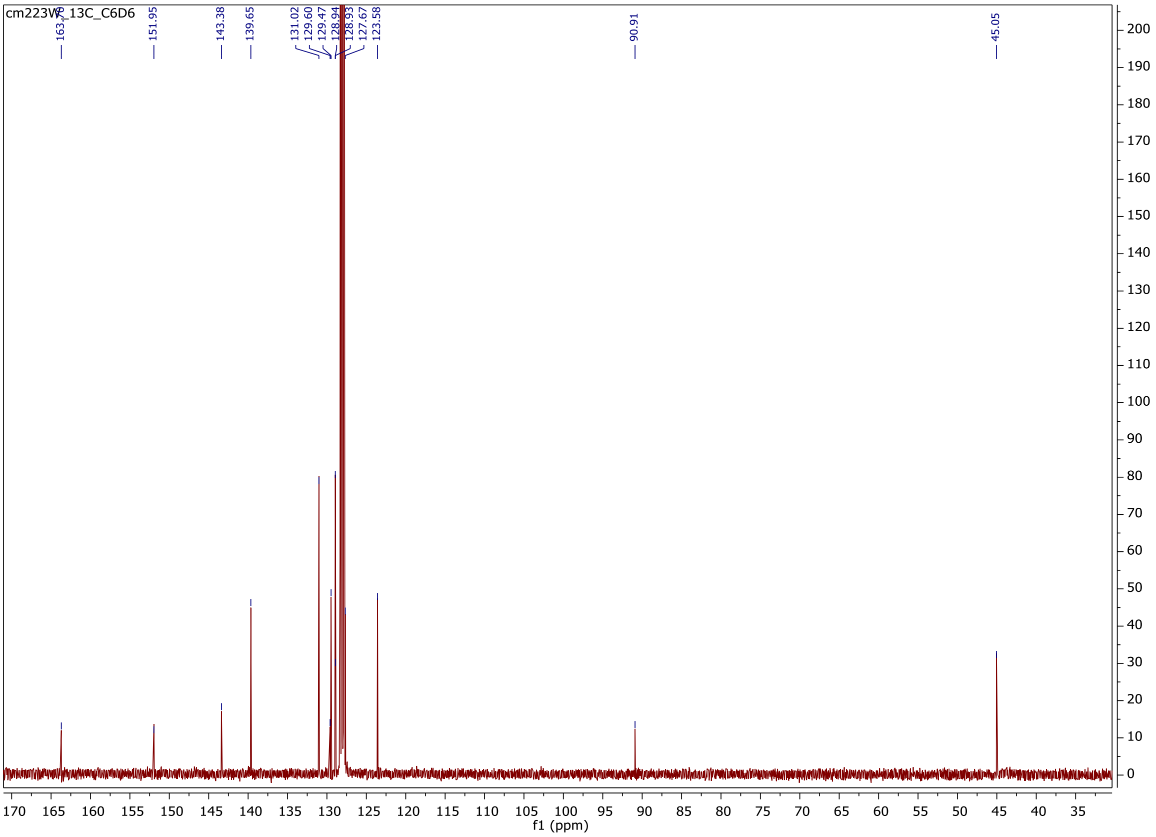

Compound **3b**

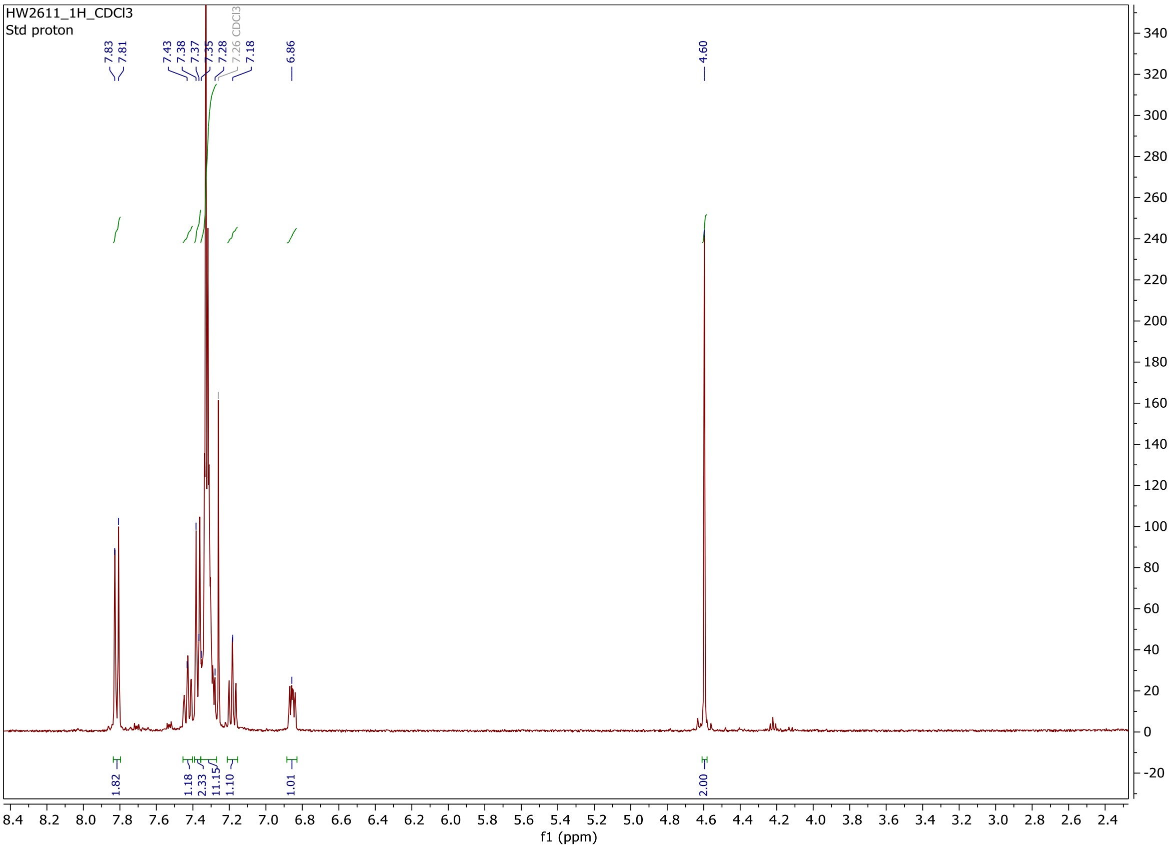
HW2611_1H_CDCl3
Std proton
7.83
7.81
7.43
7.38
7.37
7.35
7.28
7.26 CDCl3
7.18
6.86
4.60
1.82
1.18
2.33
11.15
1.10
1.01
2.00
f1 (ppm)

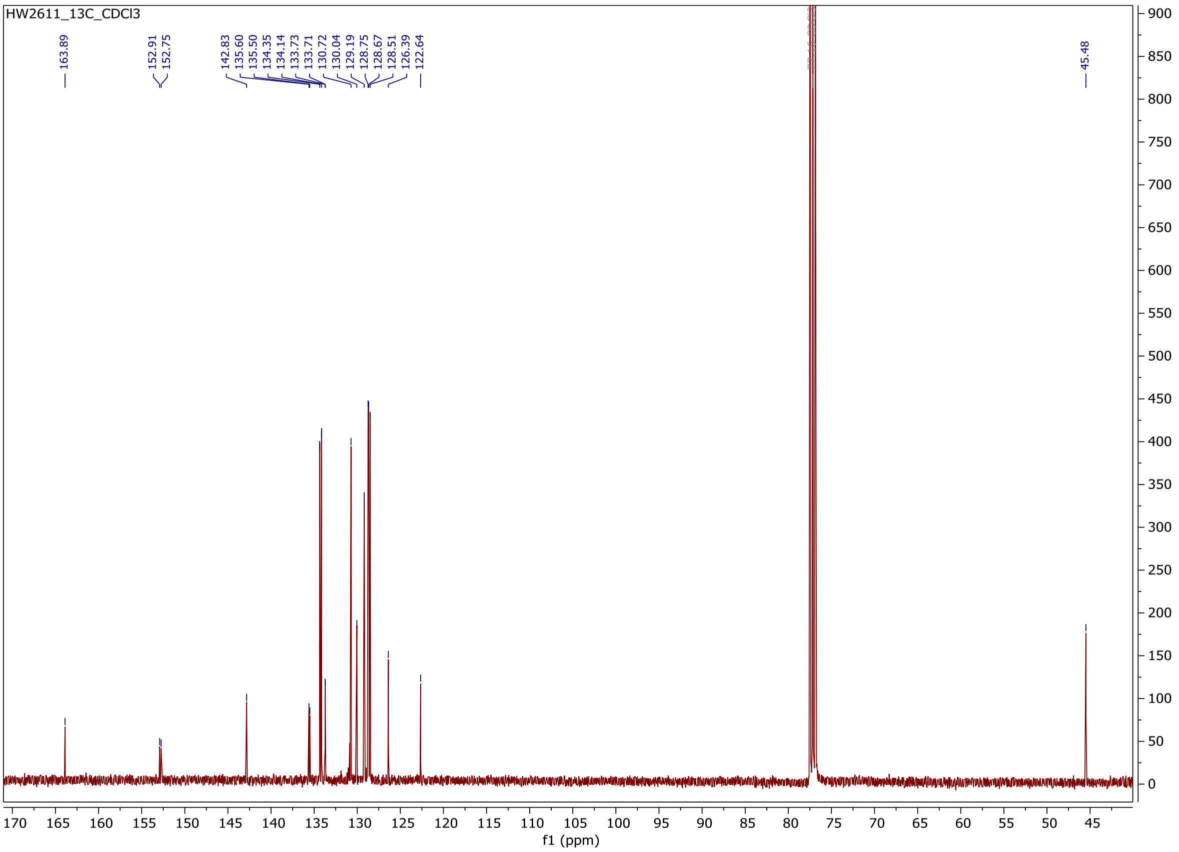
HW2611_13C_CDCl3
163.89
152.91
152.75
142.83
135.60
135.50
134.35
134.14
133.73
133.71
130.72
130.04
129.19
128.75
128.67
128.51
126.39
122.64
45.48
f1 (ppm)

Compound **3c**

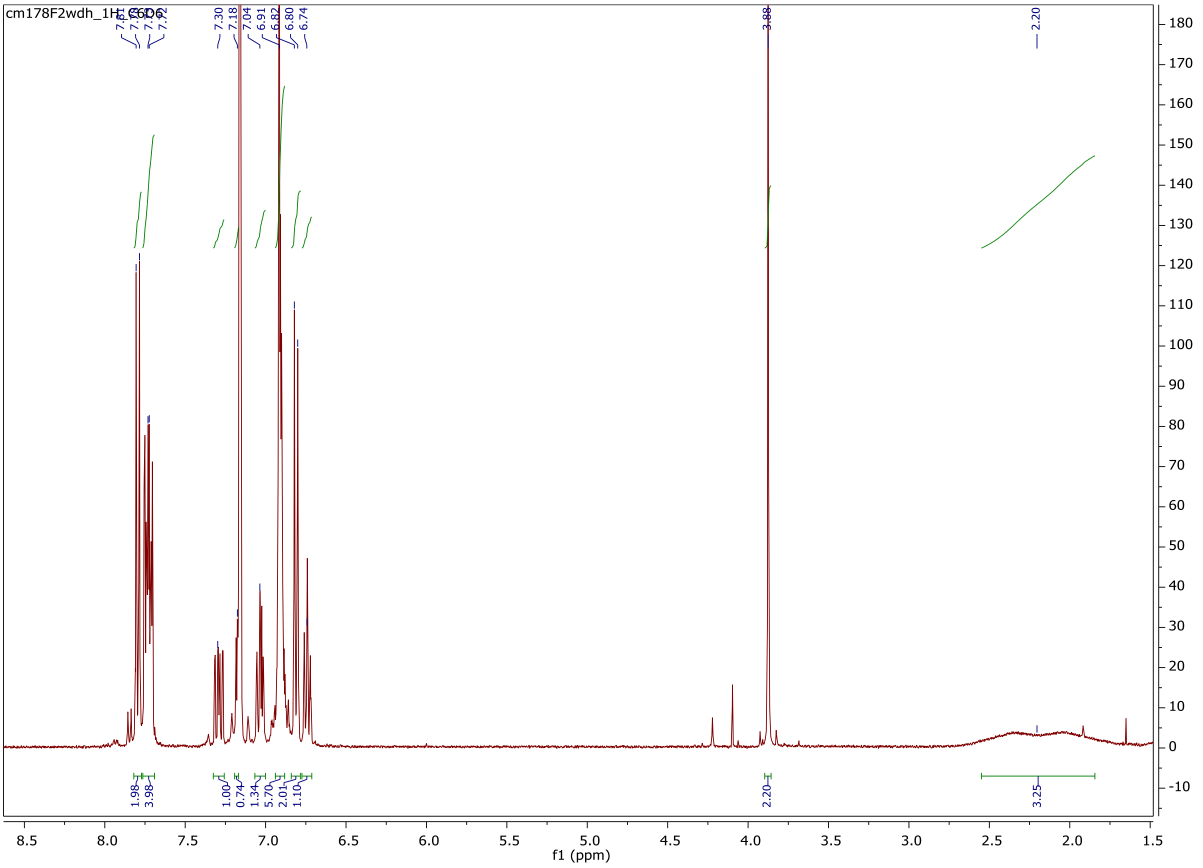

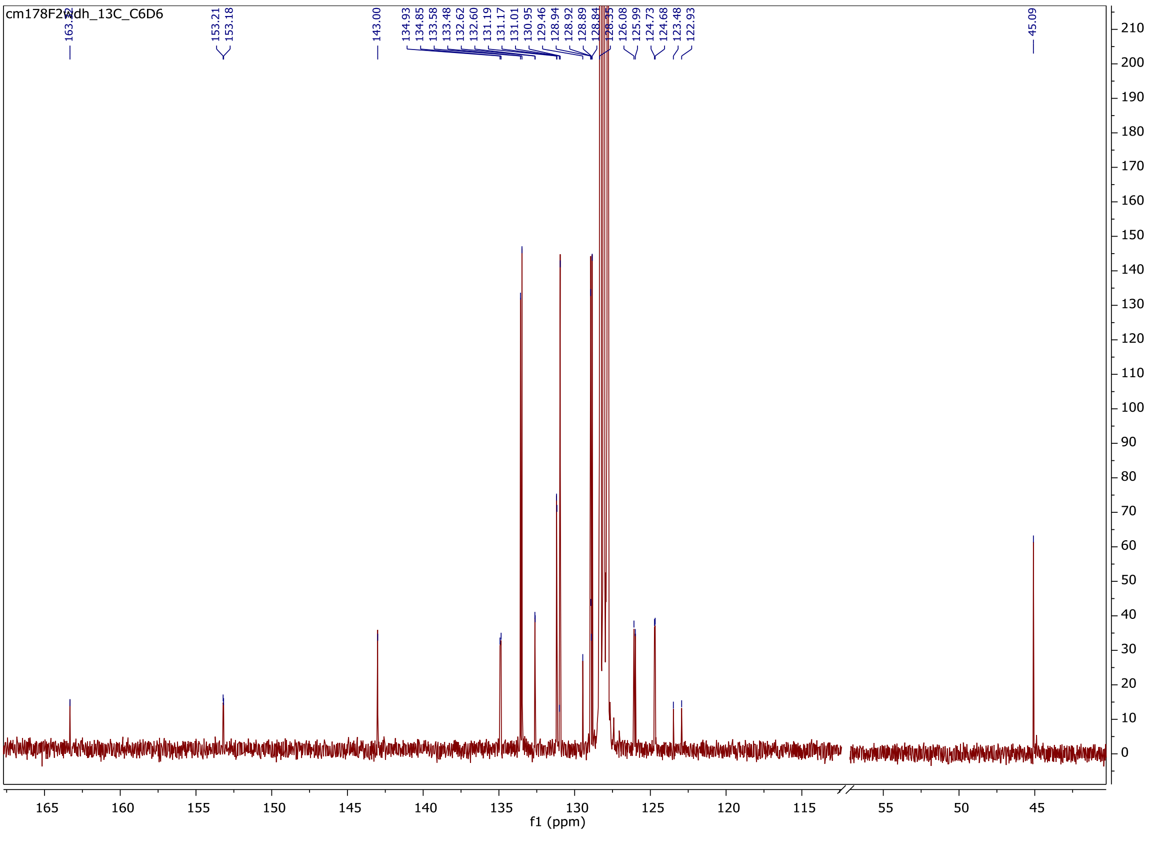

Compound **4a**

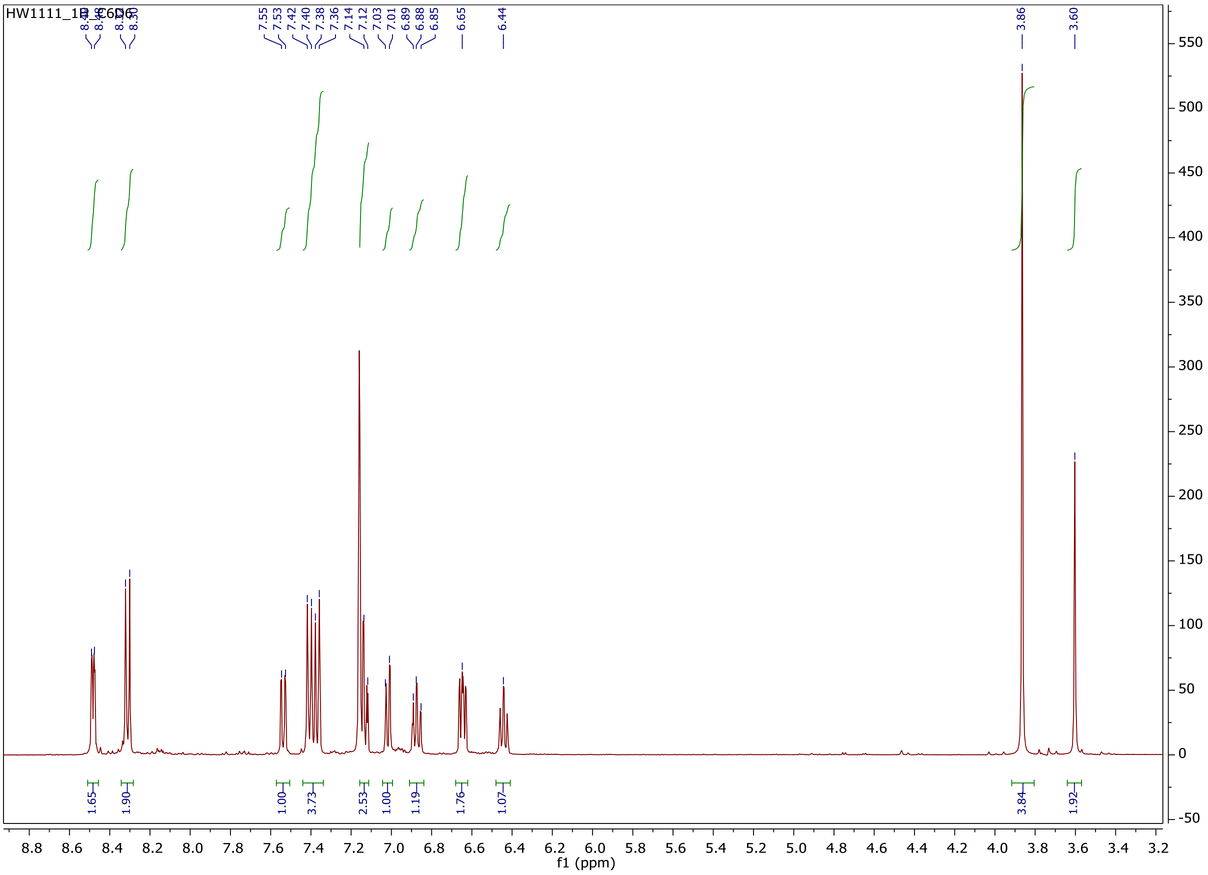

HW1111_1H_C6D6
7.55 7.53 7.42 7.40 7.38 7.36 7.14 7.12 7.03 7.01 6.89 6.88 6.85 6.65 6.44 3.86 3.60
1.65 1.90 1.00 3.73 2.53 1.00 1.19 1.76 1.07 3.84 1.92
f1 (ppm)

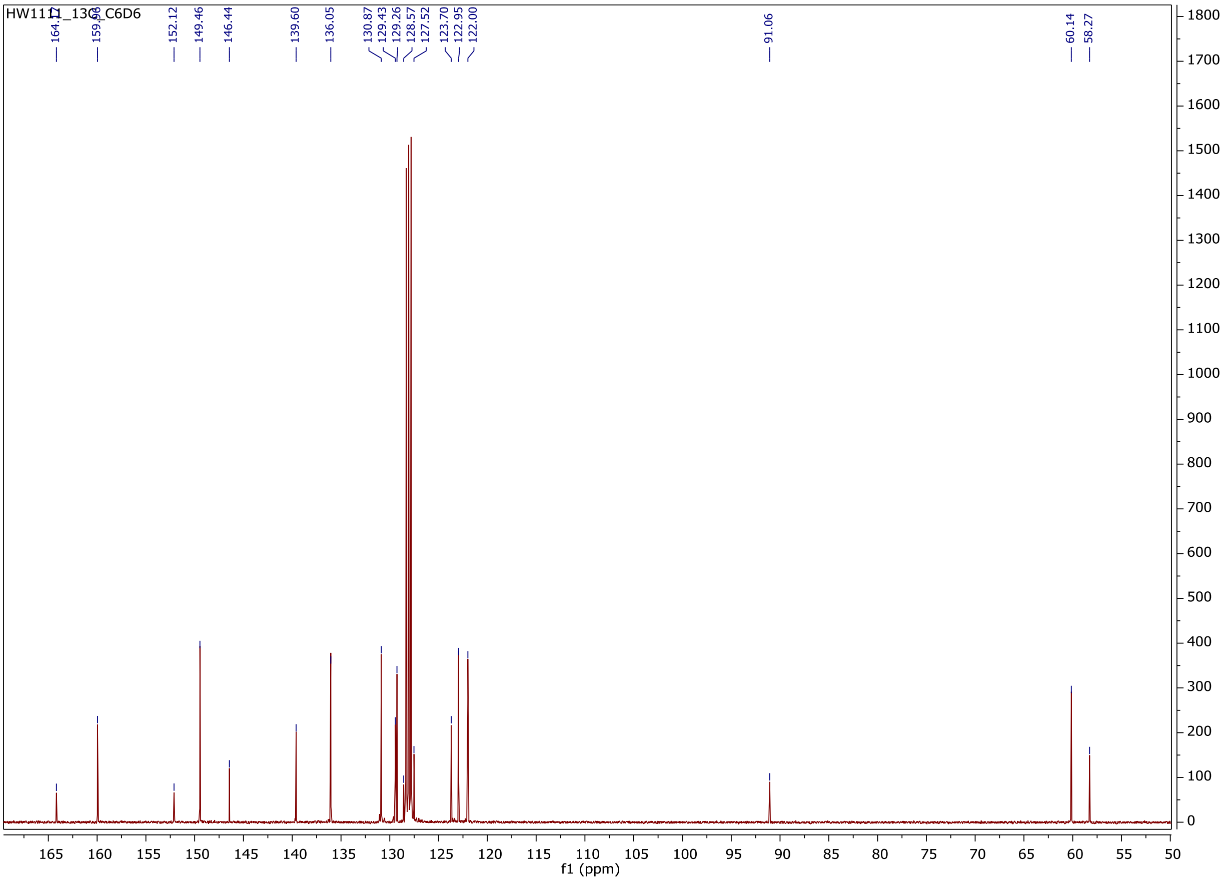

HW1111_13C_C6D6
152.12 149.46 146.44 139.60 136.05 130.87 129.43 129.26 128.57 127.52 123.70 122.95 122.00 91.06 60.14 58.27
f1 (ppm)

Compound **4b**

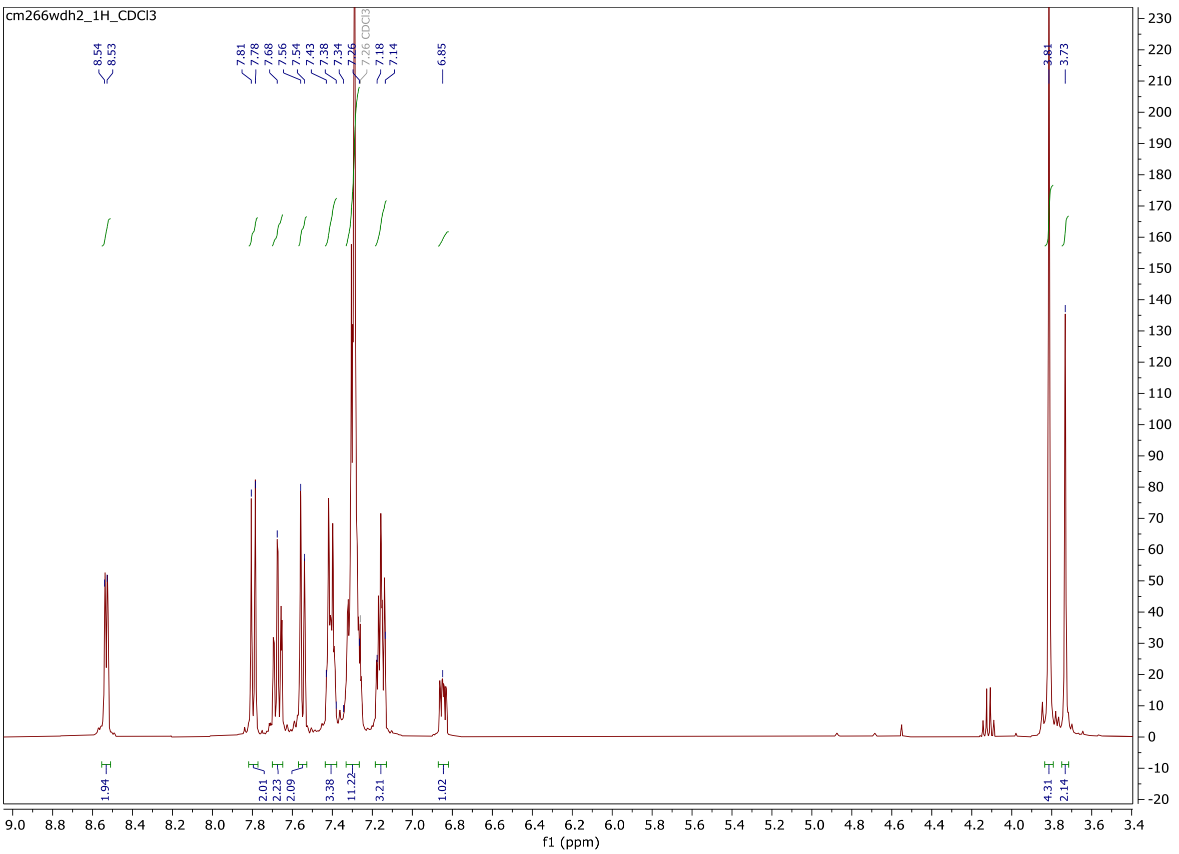

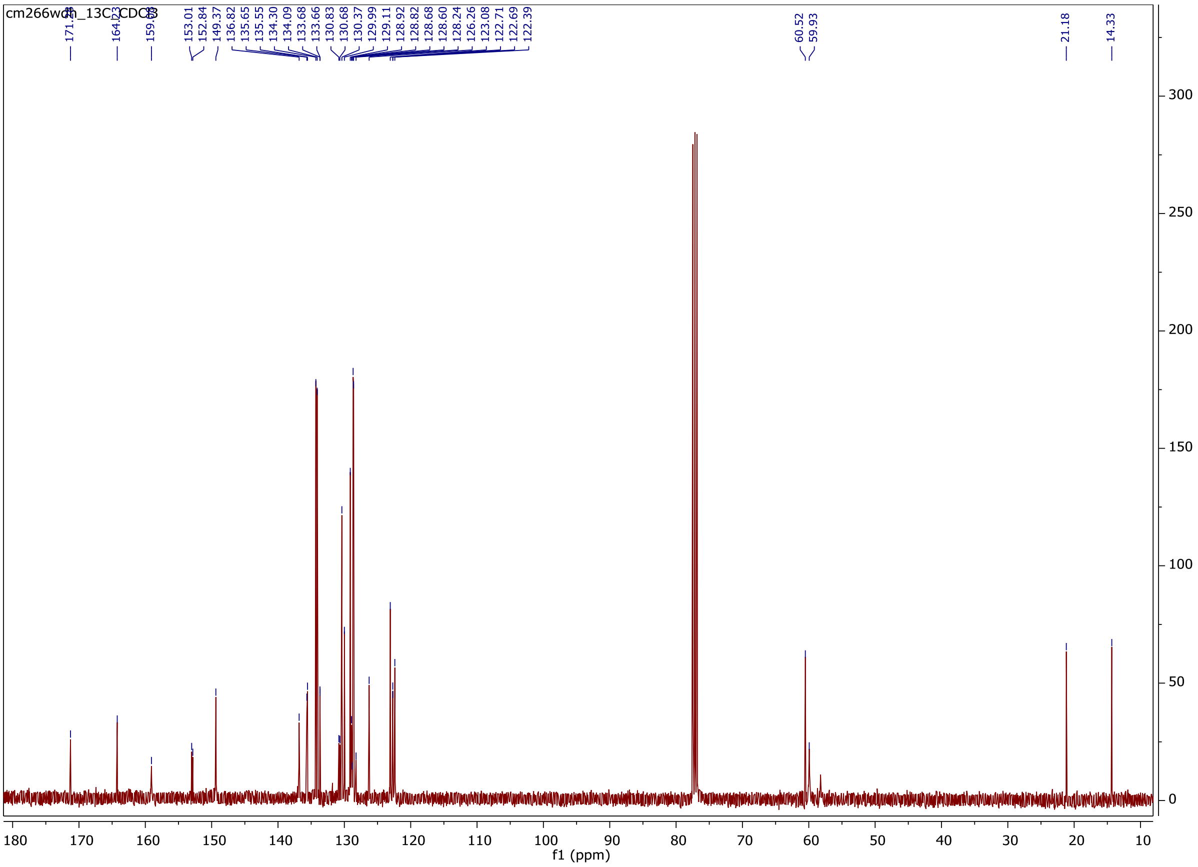

Compound 7

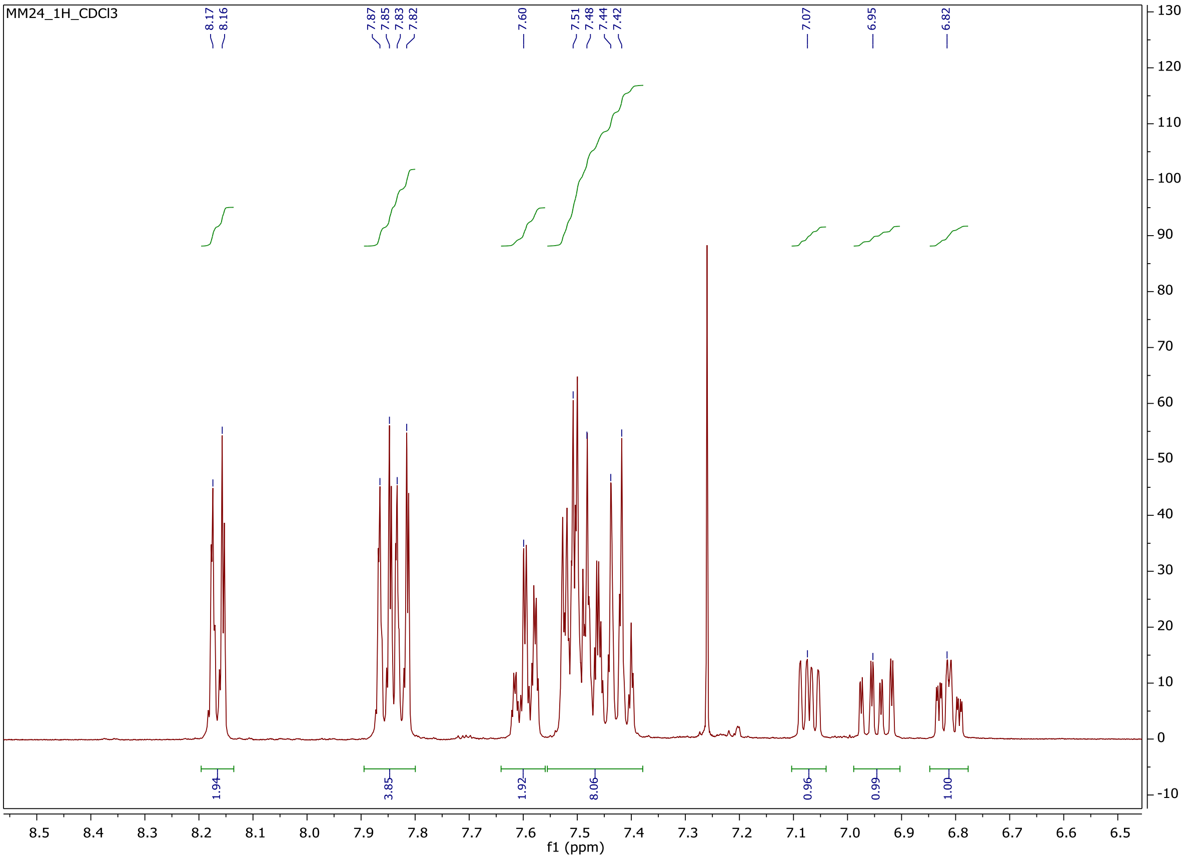
MM24_1H_CDCl3
f1 (ppm)

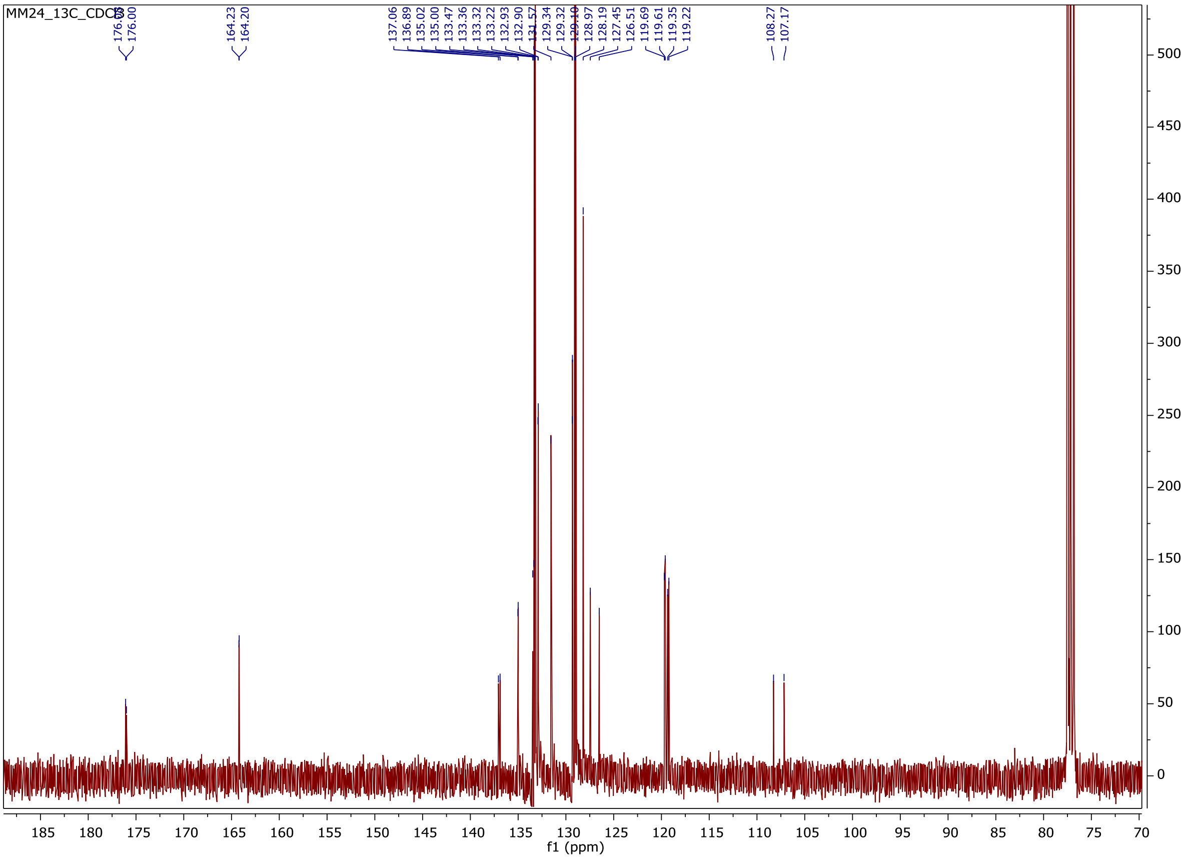
MM24_13C_CDCl3
f1 (ppm)

Compound **8**

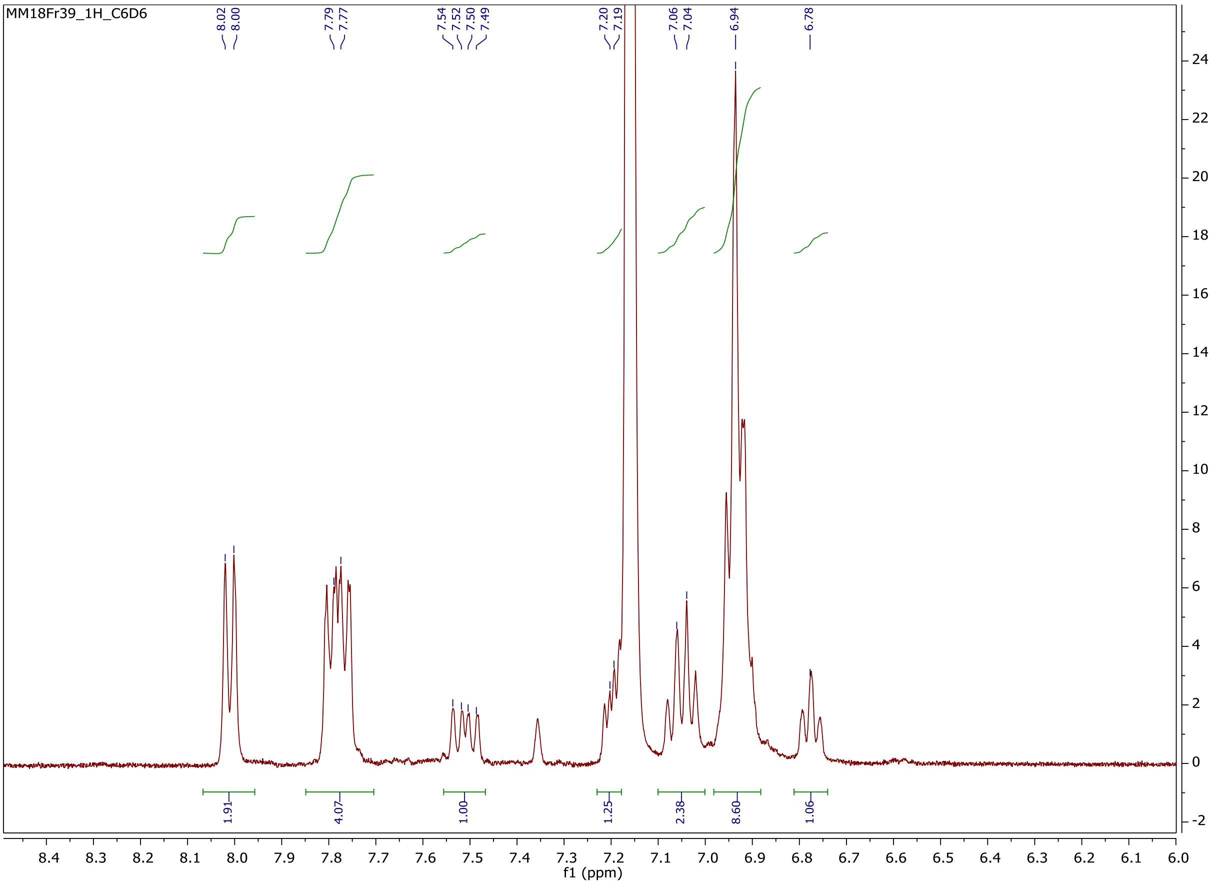

MM18Fr39_1H_C6D6
8.02
8.00
7.79
7.77
7.54
7.52
7.50
7.49
7.20
7.19
7.06
7.04
6.94
6.78
1.91
4.07
1.00
1.25
2.38
8.60
1.06
f1 (ppm)

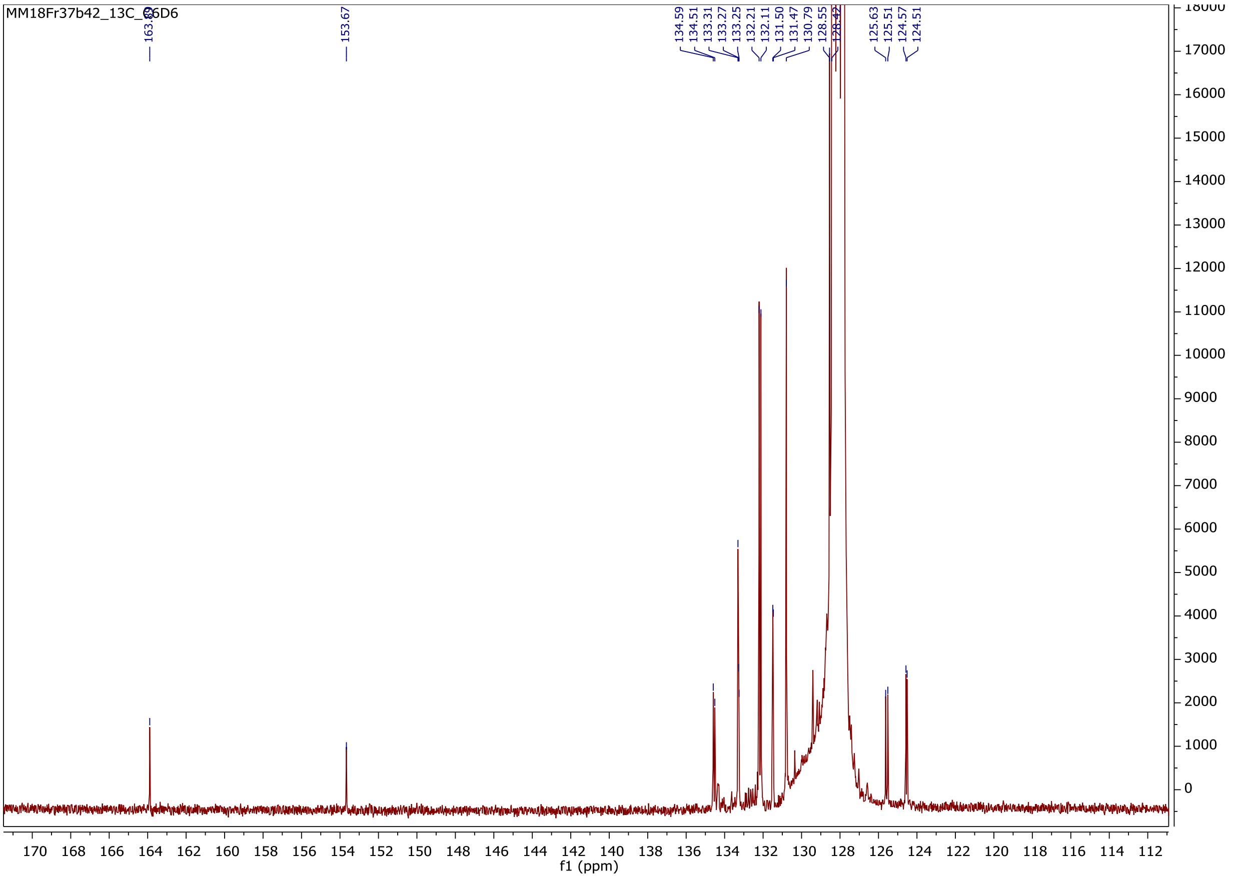

MM18Fr37b42_13C_C6D6
153.67
134.59
134.51
133.31
133.27
133.25
132.21
132.11
131.50
131.47
130.79
128.55
128.42
125.63
125.51
124.57
124.51
f1 (ppm)

fac-[Re(CO)$_3$4a]Br

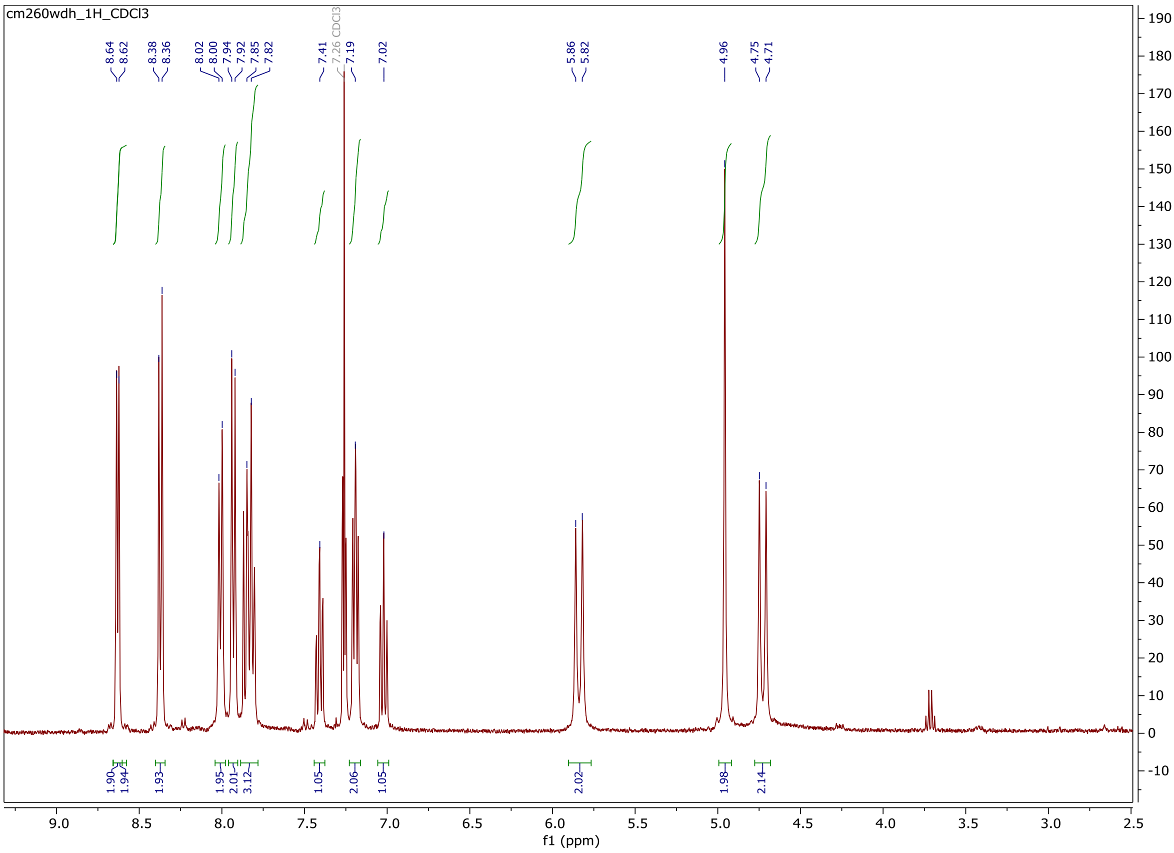

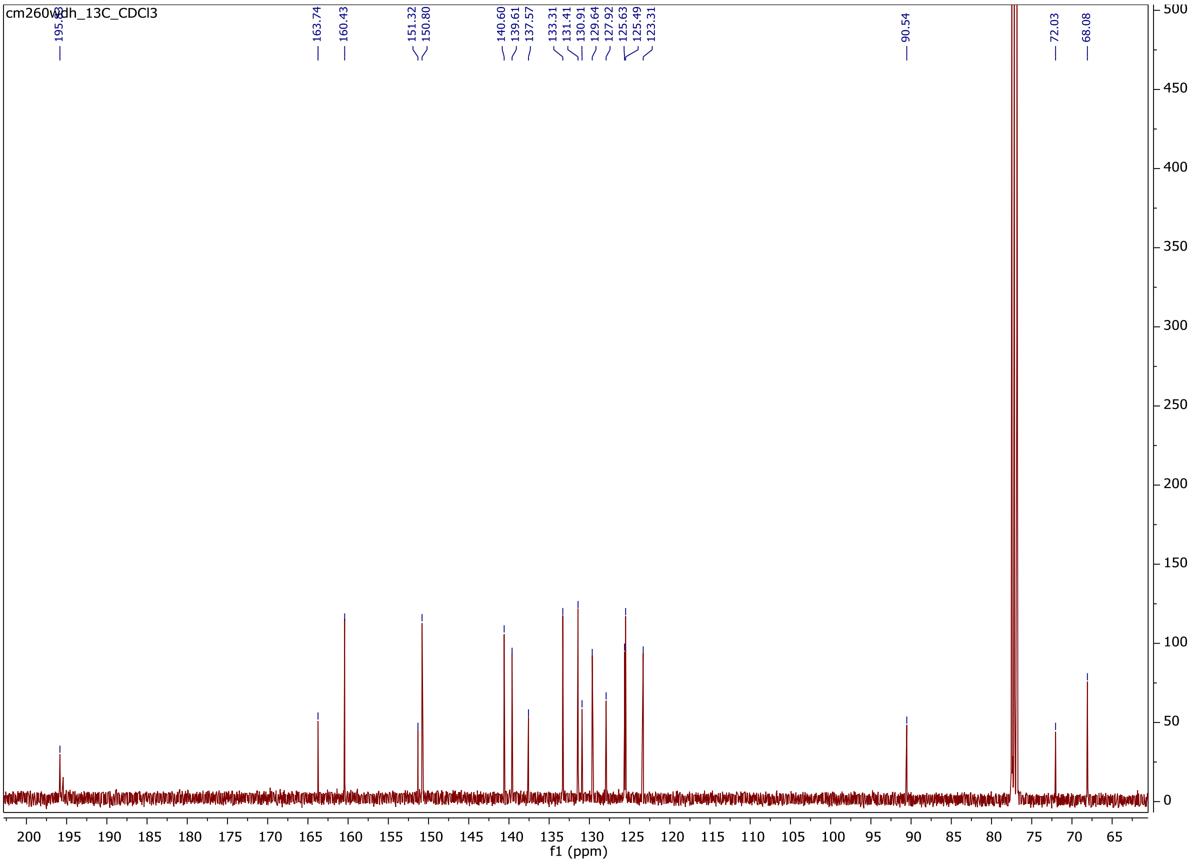

Acetonitrile-tris-carbonyl-(2-(diphenylposphino)phenolato)-rhenium(I) 9

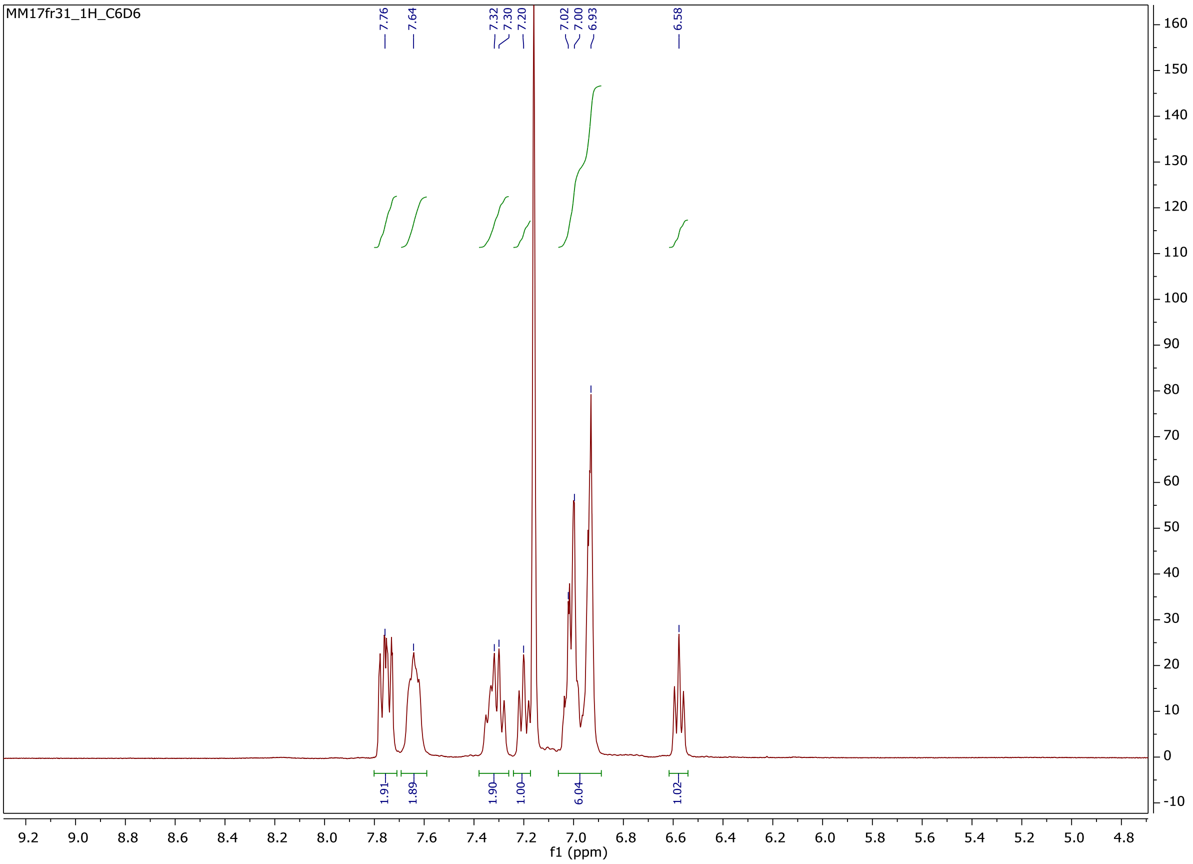

MM17fr31_1H_C6D6
7.76
7.64
7.32
7.30
7.20
7.02
7.00
6.93
6.58
1.91
1.89
1.90
1.00
6.04
1.02
f1 (ppm)

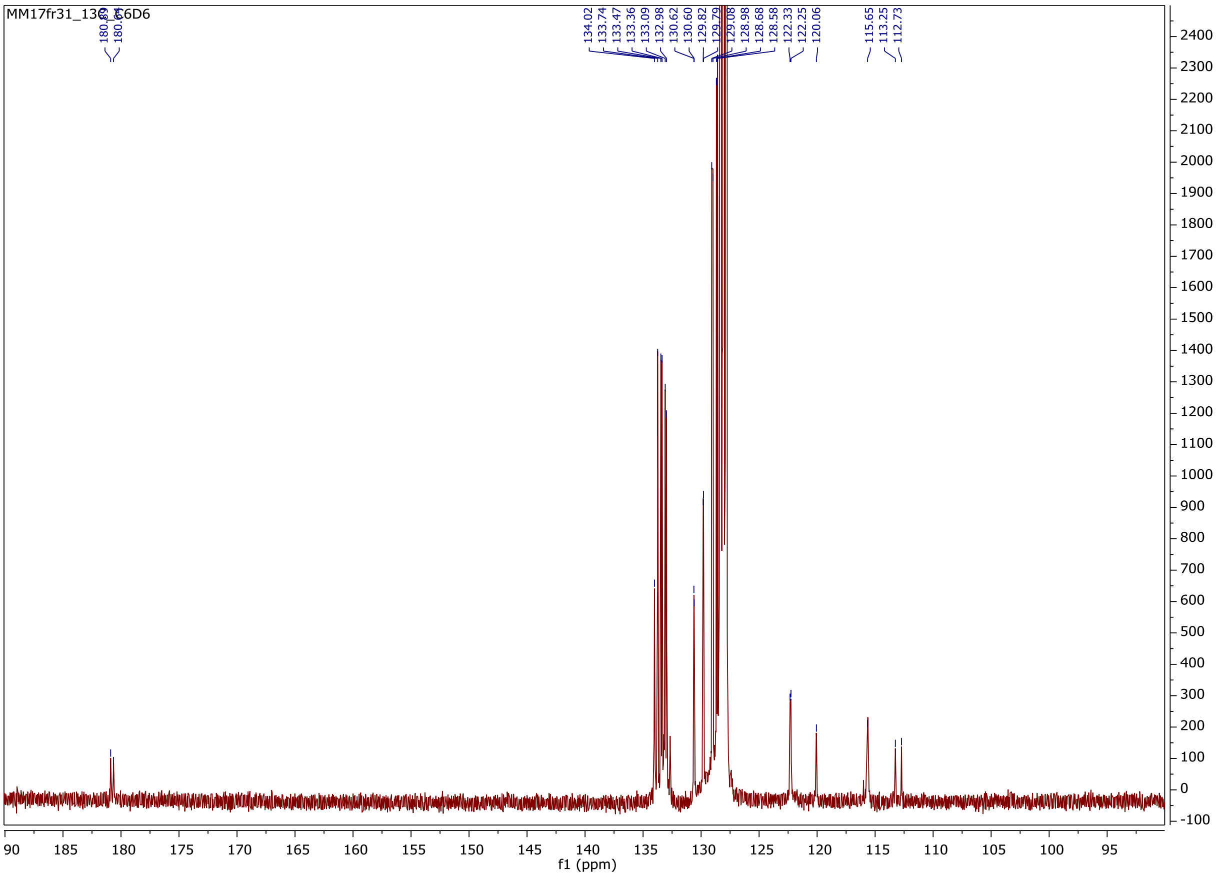

MM17fr31_13C_C6D6
134.02
133.74
133.47
133.36
133.09
132.98
130.62
130.60
129.82
129.79
129.08
128.98
128.68
128.58
122.33
122.25
120.06
115.65
113.25
112.73
f1 (ppm)

HPLC chromatograms of ligands 4a and 4b

Ligand **4a**

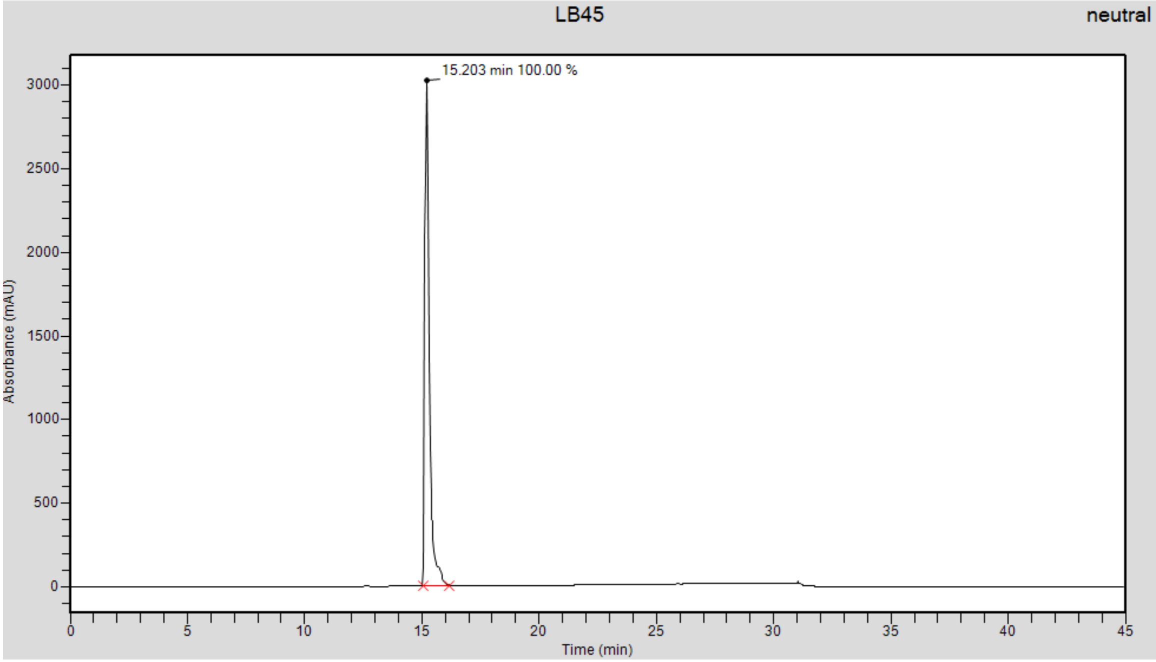

Ligand **4b**

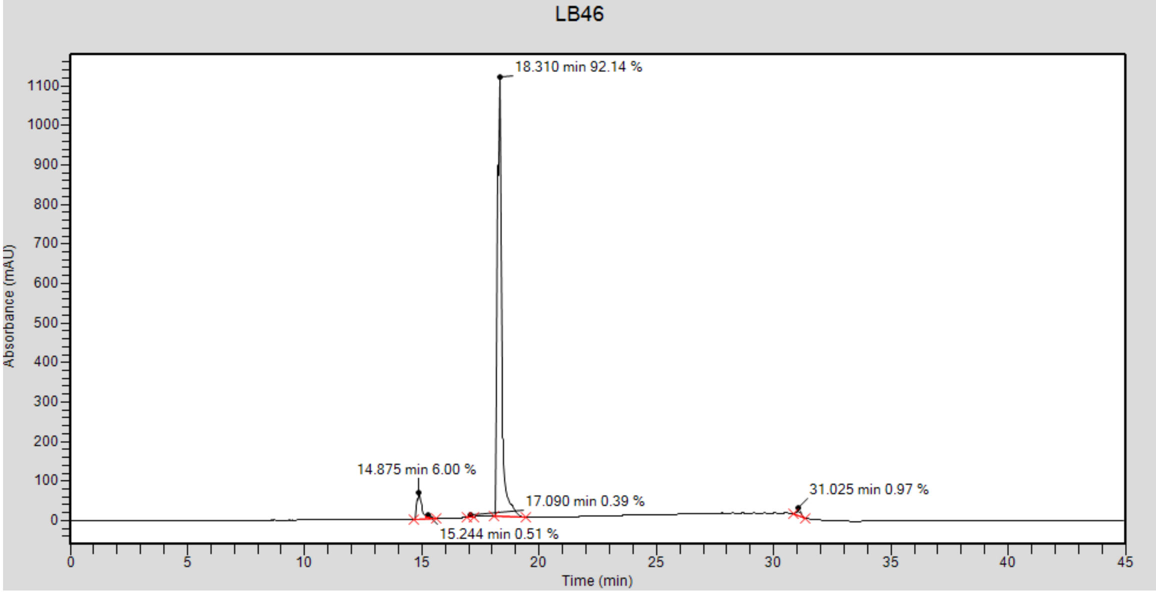

HPLC chromatogram of *fac*-[Re(CO)$_3$4a]Br

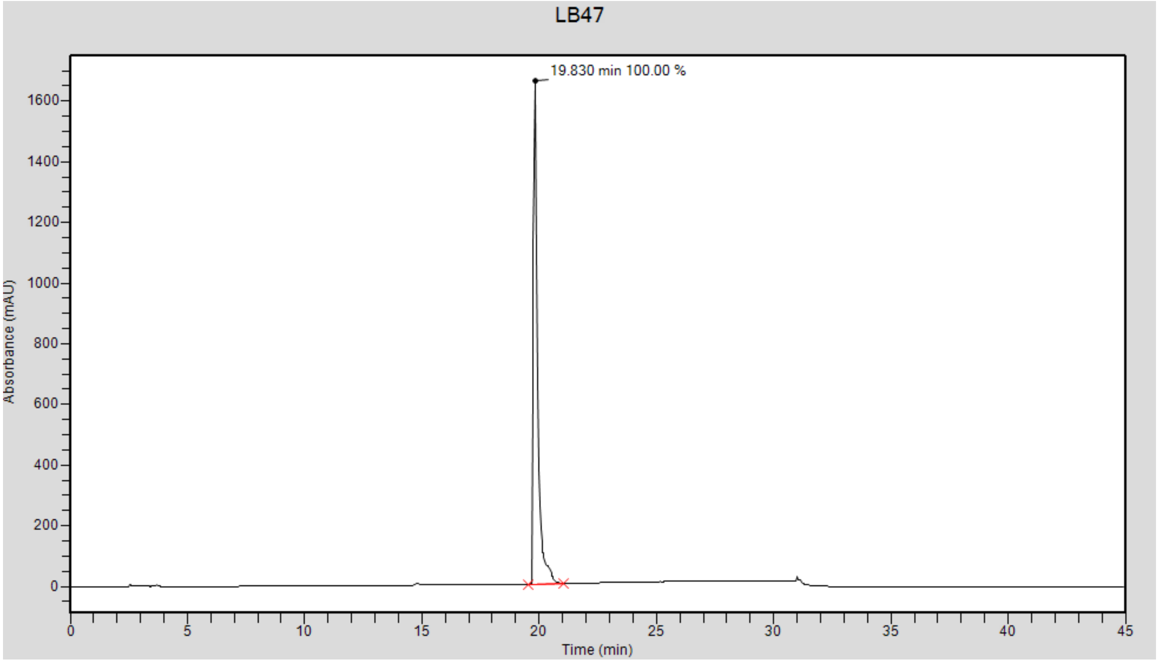

Tricarbonyl kit and ^{99m}Tc-precursor

Content: 17 mg sodium tartrate, 3.5 mg $NaBH_4$, 3.2 mg Na_2CO_3, 8.1 mg potassium boranocarbonate

Radiolabeling

- 1 ml of $Na^{99m}TcO_4$ (500 MBq) was added to the tricarbonyl kit
- 30 min @ 100 °C, 15 min brought to rt

HPLC conditions for analysis of radiolabeling

Column: Jupiter 4µ Proteo C18 90A; 4,6 x 250 mm

Solvents: (A) H_2O + 0.1% TFA / (B) acetonitrile + 0.1% TFA

Eluent: 3` 95 % (A); 20` 95 % → 5 % (A); 5` 5 % (A); 2` 5 → 95 % (A); 15` 95 % (A)

Flow rate: 1 mL/min

Radio HPLC chromatogram of *fac*-[[^{99m}Tc]Tc(CO)$_3$(H$_2$O)$_3$]$^+$

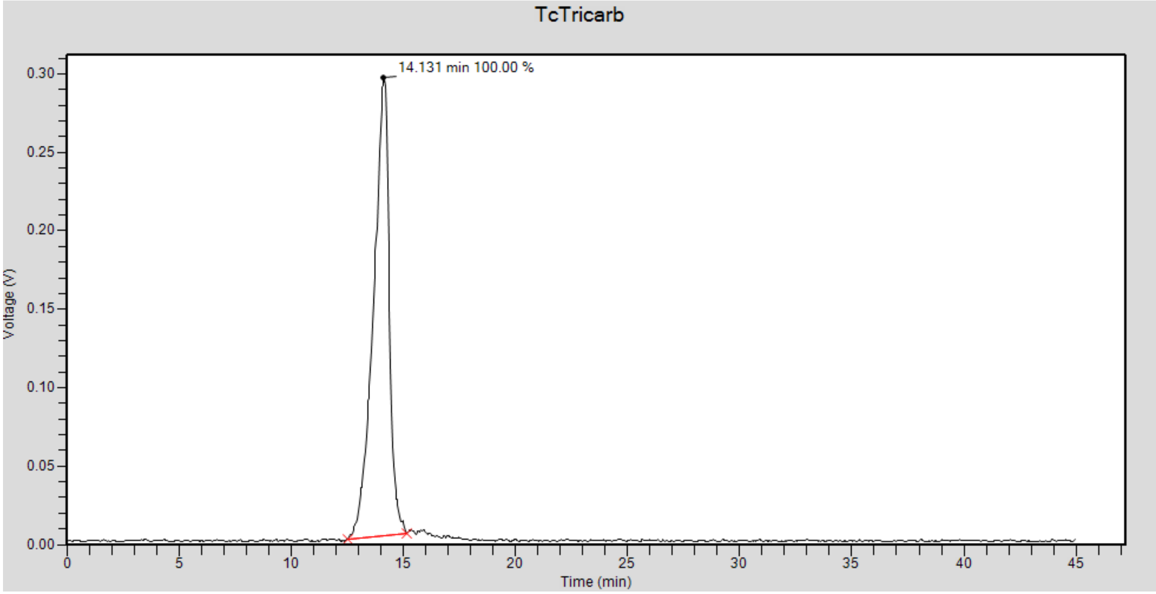

Radiolabeling procedure of ligands 4a and 4b

Radiolabeling

- addition of 500 µL 1 M MES pH 6,2 to the reaction vial containing *fac*-$[[^{99m}Tc]Tc(CO)_3(H_2O)_3]^+$
- addition of 200 µg ligand **4a** or **4b** dissolved in 200 µL of ethanol
- heated for 30 min at 100°C
- filtration

Purification

- RP cartridge (LiChrolut RP-18 (40 - 63 µm), 500 mg) loading with the reaction mixture
- wash 3 x with 2 mL of H_2O
- elution with 2 mL of ethanol

Radio HPLC chromatograms of *fac*-[[^{99m}Tc]Tc(CO)$_3$4a]$^+$

Reaction mixture

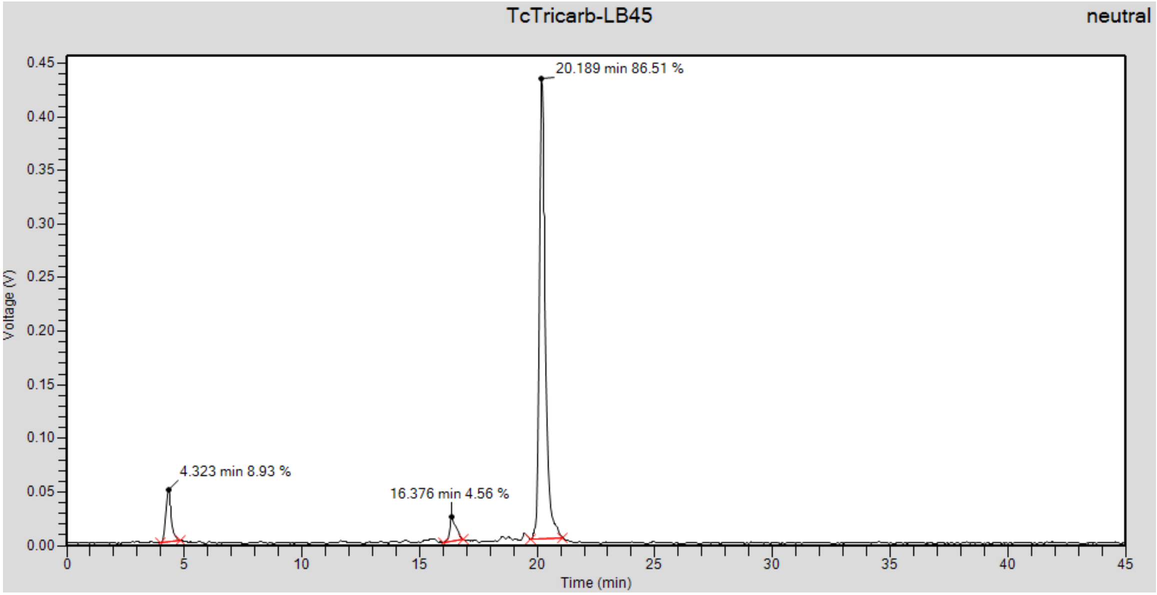

After purification

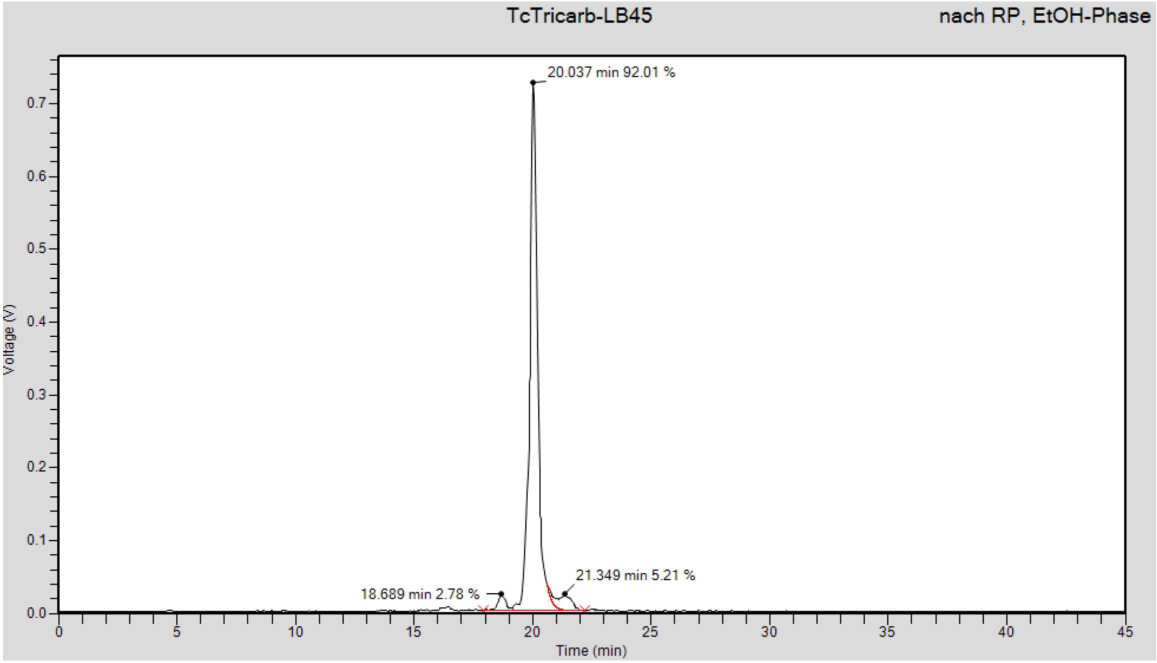

Radio HPLC chromatograms of *fac*-[[^{99m}Tc]Tc(CO)$_3$4b]$^+$

Reaction mixture

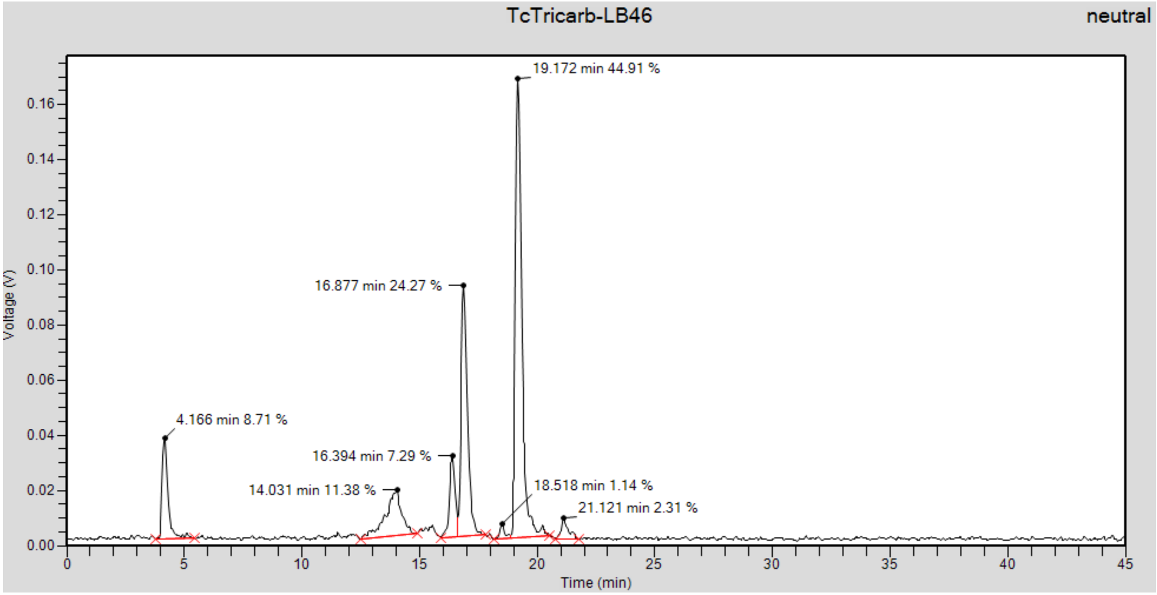

After purification

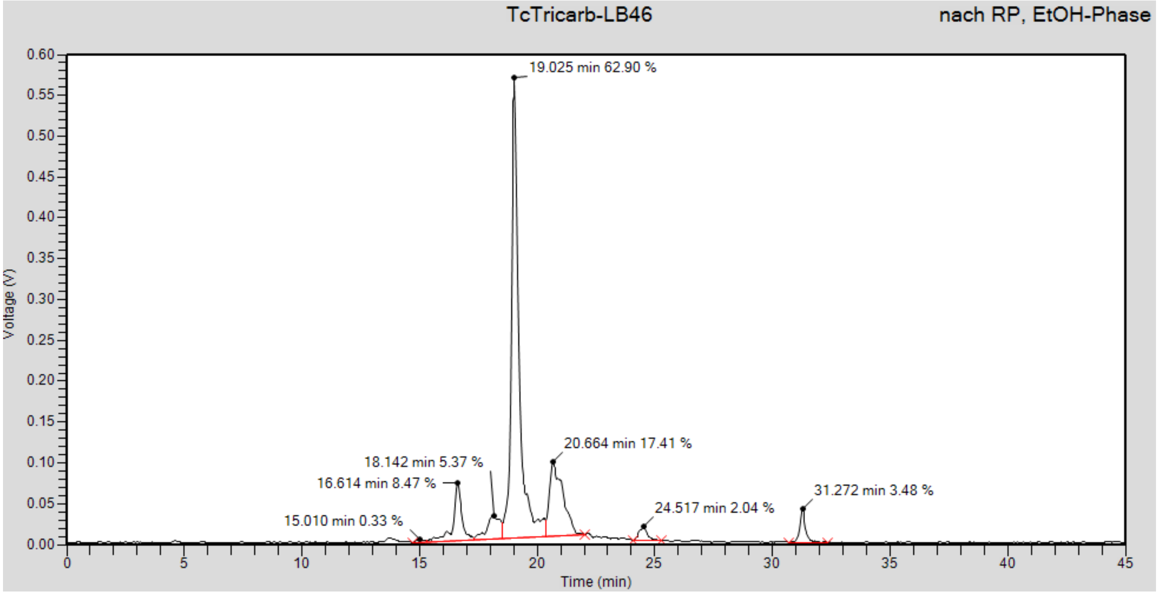

Supplement: Supplementary file 1 [file molecules-26-06629-s001.zip › molecules-1412779-supplementary.pdf]
